# A geometry-guided sizing and static force modeling protocol for compact antagonistic cable-driven joints

**DOI:** 10.1016/j.mex.2026.104219

**Published:** 2026-10-19

**Authors:** Adilet Toleshev, Sharafatdin Yessirkepov, Aibek Niyetkaliyev, Ali Kaiyrdykozha, Aiman Ozhikenova, Lashin Bazarbay, Ozhiken Assylbek, Igembay Yerbolat

**Affiliations:** a Nazarbayev University, Robotics Engineering Department, Astana, 010000, Kazakhstan; b School of Intelligent Systems, Astana IT University, Astana, 010000, Kazakhstan; c Qazaq Youth Science Hub LLP, Almaty, 050020, Kazakhstan; d Satbayev University, Department of Robotics and technical means of automation, Almaty, 050013, Kazakhstan; e Institute of Mechanics and Mechanical Engineering named after Academician U.A. Dzholdasbekov, Almaty, 050010, Kazakhstan

**Keywords:** Robotic elbow, Antagonistic actuation, Cable-driven mechanisms, Kinematic evaluation, Collision avoidance, Workspace analysis

## Abstract

This paper presents a framework for the kinematic modelling, pseudo-static force analysis, and parametric evaluation of an antagonistic cable-driven robotic joint, demonstrated on a miniature elbow prototype. Analytical transformation and equilibrium models were formulated for a dual-cable architecture to resolve trade-offs between range of motion, actuation force, and cable displacement. Parametric sweeps across key structural dimensions identified a preferable base-to-platform distance (h=240mm), enabling collision-free articulation across a $135^{\circ }$ range of motion. Evaluating the cable holder radius (r) designated r=40mm as the preferred configuration, satisfying geometric constraints while restricting cable displacement to limit motor spool size. Hardware-in-the-loop testing validated the analytical models across single- and dual-cable antagonistic configurations. Kinematic tracking yielded RMSEs of 3.82 mm (peak error: 8.92 mm) for flexion and 1.46 mm (peak error: 3.56 mm) for extension. Static force validation achieved RMSEs of 0.51 N (peak error: 1.55 N) in single-cable testing and 0.38 N (peak error: 0.48 N) during dual-cable antagonistic operation. The proposed methodology offers a guideline for developing compact, lightweight, single-degree-of-freedom cable-driven robotic systems.

## Graphical abstract

**Figure:**
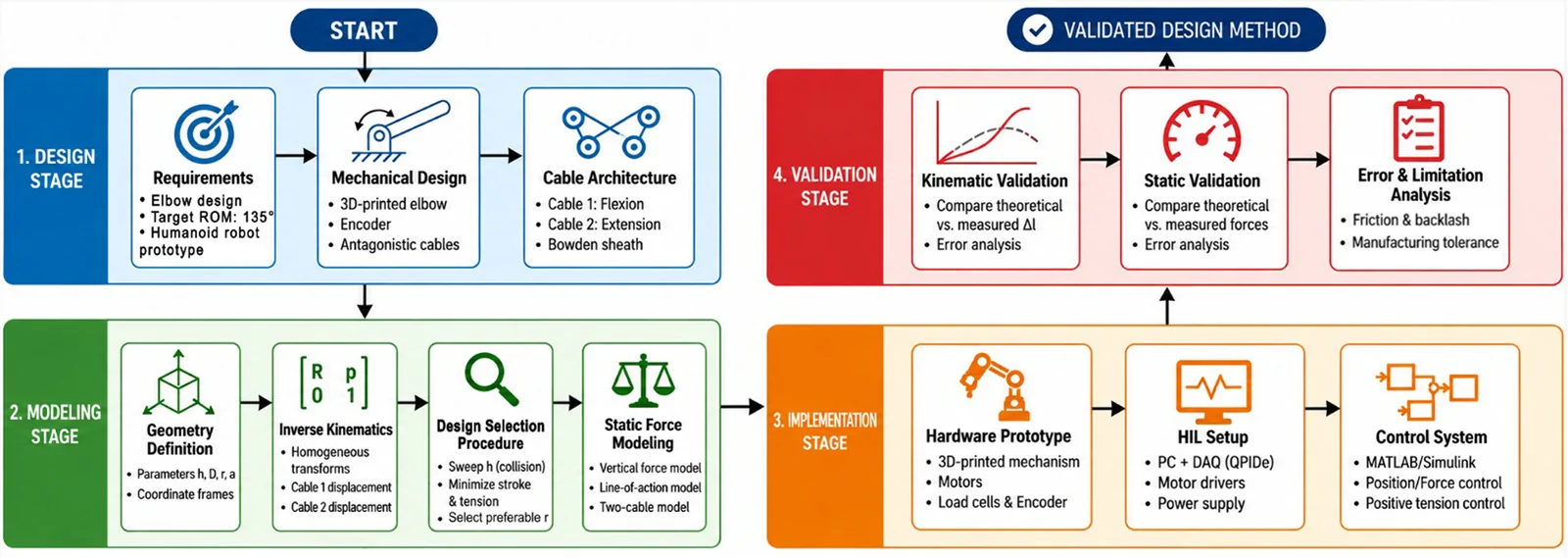

## Specifications table

**Table:** 

**Subject area**	Engineering
**More specific subject area**	Cable-driven mechanisms; Mechatronic system design; Robotics
**Name of your method**	Geometry-guided modeling and validation method for a compact antagonistic cable-driven elbow joint
**Name and reference of original method**	Not applicable. This article describes an integrated method developed for a compact 1-DOF antagonistic cable-driven elbow joint. The method combines established cable-driven mechanism principles, inverse kinematic modeling, static equilibrium analysis, collision-aware cable routing, and hardware-in-the-loop validation for a 1-DOF antagonistic cable-driven robotic elbow prototype.
**Resource availability**	CAD files, MATLAB/Simulink models, and raw experimental data associated with this study are available in the Supplementary Material section.

## Background

Cable-driven parallel robots (CDPRs) employ flexible cables routed from remotely located actuators to transmit forces to a moving platform or joint, reducing moving mass and inertia while enabling large workspaces and high payload-to-weight ratios [1,2]. Because cables can only pull and never push, cable-driven mechanisms must preserve positive cable tension and wrench feasibility throughout the prescribed motion, making workspace analysis and tension management central design requirements [3].

Antagonistic cable-driven joints, in which two opposing tendons regulate motion and stiffness, offer a biologically inspired actuation principle that supports safer and more adaptable interaction with the user [4].

At the mechanism-design level, anchor and routing geometry strongly govern the performance of such devices. In CDPRs generally, cable attachment locations determine the wrench-feasible and interference-free regions as well as the required cable forces [5]. Thus, cable attachment is not only a layout choice but an important design variable affecting feasible workspace and force transmission. Similar trade-offs between cable direction, joint workspace, and tension efficiency arise in upper-limb robots [6]. For elbow-assistance systems specifically, the distance between cable anchor points and the elbow joint changes cable tension, joint reaction forces, and generated moments, directly affecting range of motion (ROM), and performance [7].

Beyond geometry, antagonistic cable-driven joints require coordinated tension management to remain taut and controllable: tension optimization keeps forces within feasible positive limits while producing smooth profiles [8], co-activation and elastic elements regulate equilibrium position and stiffness [9], and hybrid force–position control combines trajectory tracking with tension regulation to avoid slack or over-tension [10].

Recent studies have further considered tension constraints, disturbances, and transmission effects in cable-driven robots. Сable-driven robot control was investigated under explicit cable-tension constraints in [11], while an optimization-based control approach for precision trajectory tracking of cable-driven parallel robots was proposed in [12]. In another study, the pulley friction and other disturbances were considered in trajectory tracking [13]. In compact elbow devices, position control alone is therefore insufficient — cable preload and tension feedback must be part of the control objective.

Feasible elbow motion also depends on collision-free cable routing. Multiple cables sharing a confined workspace create cable–cable, cable–platform, and cable–link interference risks [14]. Due to these restrictions, cable layout and collision prevention are key design considerations for compact cable-driven mechanisms. These interconnected challenges—anchor placement, link clearance, routing, and tensioning—are magnified in compact single-DOF elbow joints, leaving little margin to resolve them independently.

Multi-objective cost functions have increasingly been adopted to resolve geometric trade-offs in cable-driven parallel robots (CDPRs). The use of a weighted-sum cost function was demonstrated to evaluate and select optimal geometric configurations in CDPR design [15]. Similarly, the workspace volume was highlighted as the main evaluative criterion when synthesizing CDPR geometries [16]. However, these methodologies are tailored either to distinct spatial architectures or overconstrained setups relying on complex hybrid position–force control schemes. Extending multi-objective cost-function evaluation to compact, single-degree-of-freedom antagonistic joint mechanisms—where link clearance, cable travel, and static tension must be balanced concurrently—remains limited.

Previous studies demonstrate that cable attachment geometry, tension evaluation, antagonistic actuation, force–position control, and interference avoidance have each been investigated in cable-driven mechanisms [5,[8], [9], [10], [11], [12], [13], [14]], while recent works have further addressed tension constraints, optimization-based control, disturbances, and pulley friction [[11], [12], [13]]. However, these studies generally consider different aspects of the mechanism separately and often treat anchor and routing geometry as fixed parameters during synthesis. To bridge this gap, the present study introduces a systematic geometric parameter selection procedure.

A systematic treatment of how base-to-platform spacing and cable-holder placement jointly determine workspace, collision-free ROM, cable displacement, and actuation force in a compact antagonistic configuration remains limited. In the present work, these geometric parameters are explicitly varied and evaluated using inverse kinematics and static force modeling, and the resulting trade-offs are incorporated into cost functions for selecting the geometric design.

This method addresses that gap by providing a geometry-guided sizing and force-modeling selection procedure — together with a 3D-printed antagonistic cable-driven elbow mechanism for its experimental validation — that other researchers can apply when designing compact, single-DOF cable-driven joints.

## Method details

### Mechanical design and parametric sizing

The initial geometric boundaries for the mechanism were scaled based on the proportions and workspace constraints of a humanoid robot elbow joint mechanism with an antagonistic cable-pair design. To establish a realistic structural envelope, the base-to-platform distance h between the upper and lower arm was systematically defined and evaluated across a range of 160 mm to 400 mm (2D to 5D for the base and platform diameter D=80 mm), as depicted in Fig. 1. Evaluating this range revealed distinct boundaries: at h=160mm, structural interference causes mechanical binding, restricting rotation to $\theta \approx 126^{\circ }$ and preventing full articulation. Extending the base-to-platform distance to 400mm eliminates collision throughout the $135^{\circ }$ range of motion, but significantly increases required cable displacement ($\Delta l_{1}>320\;\text{mm}$) and peak tension. Based on this parametric sweep, h=240mm (3D) was chosen as the balanced design point, providing full collision-free movement while keeping cable travel and actuator demands within efficient limits. Similarly, the lower-cable holder radius *r* was parameterized within a representative domain of 40 mm and 55 mm to match typical cross-sectional dimensions of humanoid joint housings. A lower bound of r=40mm was selected to satisfy the geometric criterion (r≥D/2), preventing cable and wall collisions during joint articulation while maintaining a manageable cable stroke ($\Delta l_{2}\approx 88.6\;\text{mm}$) for standard motor spools.

**Fig. 1:**
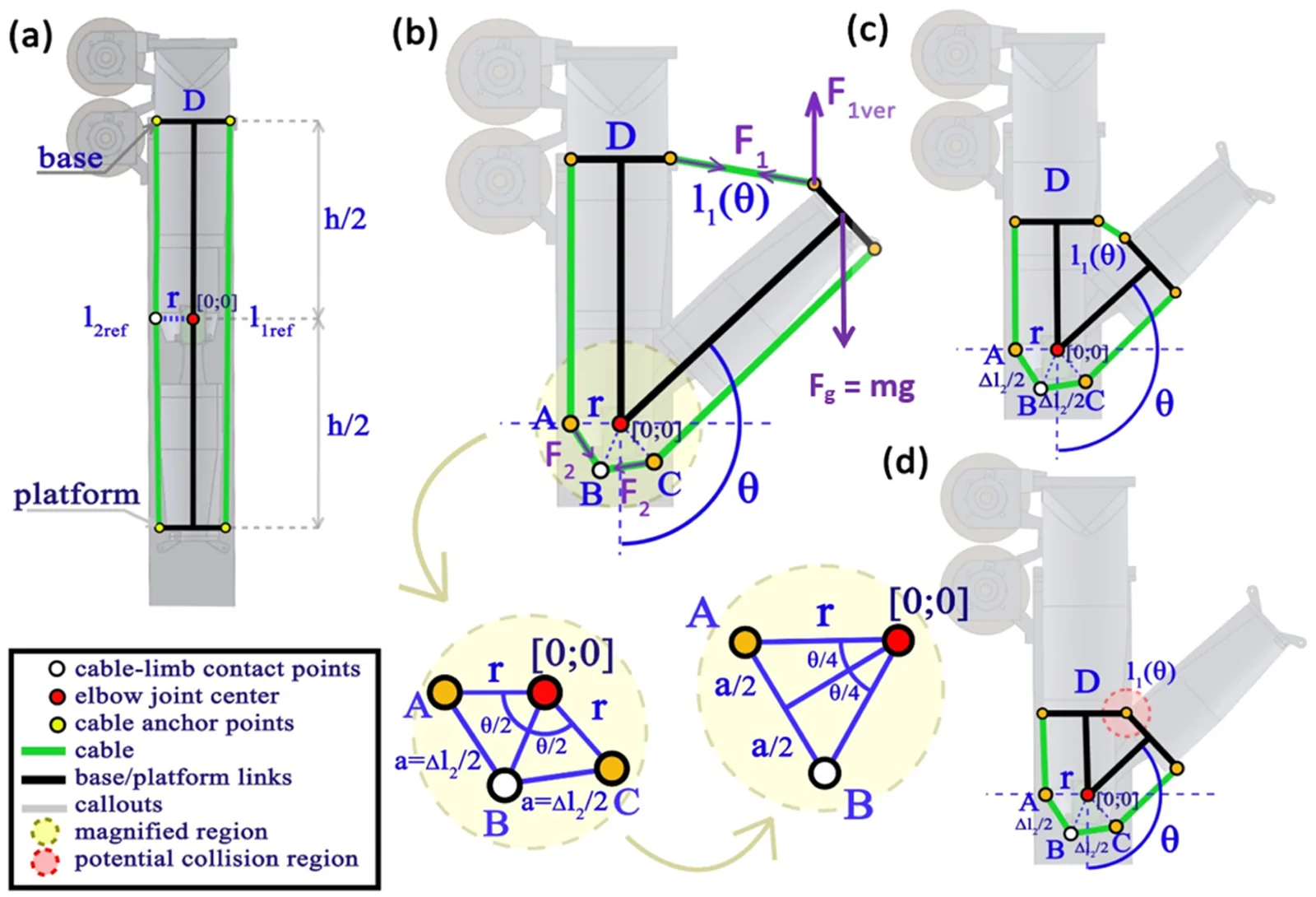
Kinematic and static behaviors of the forearm and upper-arm mechanism for different values of the base-to-platform distance h under a maximum flexion angle of 135°: (a) Neutral home configuration defining the geometric parameters h, D, a, and r; (b) h = 400 mm; (c) h = 240 mm; (d) h = 160 mm.

The upper and lower arm sections are linked by a single revolute joint (central pivot) equipped with a position encoder. Consequently, the central joint angle, alongside the known platform geometry, defines the position, velocity, and orientation of the moving platform (Fig. 2).

**Fig. 2:**
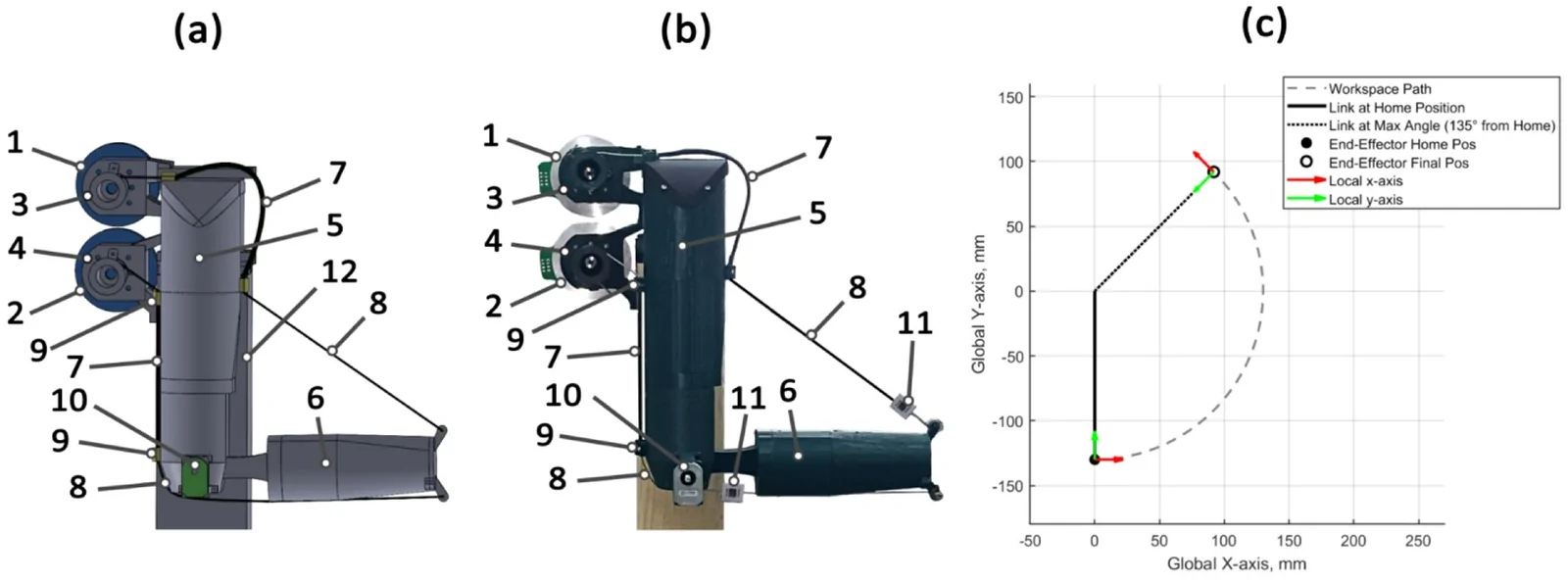
Cable-driven elbow joint mechanism featuring a 90°-rotated Bowden-cable transmission: (a) CAD model of the proposed assembly; (b) Photograph of the constructed 3D-printed prototype mounted on a benchtop structural test stand (representing a humanoid robotic arm framework), and (c) planar kinematic model, illustrating the end-effector trajectory through the workspace from neutral extension ($0^{\circ }$) to peak flexion ($135^{\circ }$).

Component breakdown: 1, 2— Upper and lower actuation motors; 3, 4 — Motor mounting brackets; 5 —Upper-arm module/proximal arm support structure (Fixed base part); 6 — Forearm link assembly (Moving Platform/End-effector part); 7 — Bowden cable sheath; 8 — 2.5-mm steel actuation cable; 9 — Bowden sheath anchoring points; 10 — Elbow joint position encoder; 11 — Inline cable tension sensor/load cell; 12 — Main support frame.

The target range of motion (ROM) is 135° of flexion from a fully extended home position (0°). The platform is mechanically restricted from rotating past the home position in the opposite direction, mirroring the unidirectional flexion behavior of the biological elbow.

To replicate this design:

- Fix the upper-arm (base) and forearm (platform) link lengths to match the target anthropomorphic proportions (h=240mm and r=40mm).
- Define the home position as full extension (0°) and the target flexion limit (135°) as the upper bound of the design ROM.
- Mount a single-axis position encoder coaxially with the revolute joint to obtain platform kinematics without distal sensors.

### Antagonistic dual-cable actuation architecture

Actuation is delivered by two cables driven by two independent motors mounted proximally on the base, keeping the distal mass low (Fig. 2). One cable (Cable 1, upper/right) pulls the platform toward flexion; the second (Cable 2, lower/left) pulls in the opposing direction and is responsible for maintaining tension and enabling extension. This antagonistic pair keeps the mechanism kinematically constrained and responsive over the full ROM, since at any instant one cable can always be tensioned against the other rather than relying on gravity.

Because the left (lower) cable's straight-line path would intersect the central joint at high flexion angles, a Bowden-cable sheath with fixed anchoring points is introduced to route the lower cable around the joint without collision (component 7, 9 in Fig. 2). The sheath serves as a passive guide operating under a simplified fixed-length routing assumption: it maintains a constant geometric distance between its two mounting points and resists the compressive reaction generated when the inner wire is tensioned, allowing the analytical model to approximate displacement transmission along the curved housing. To replicate this architecture:

1. Assign one cable to act on flexion and the second, routed on the opposite side of the joint, to act on extension/tensioning.
2. Identify the rotation range over which the non-primary (left) cable's straight-line path would intersect the joint housing.
3. Insert a Bowden sheath (or equivalent rigid-length guide) with fixed anchor points to carry that cable around the joint over the conflicting range; verify by inspection or simulation (Section 4 below) that the routed path stays clear of all moving links across the full ROM.

### Geometric parameterization and kinematic modelling

The mechanism is modelled as a planar Cable-Driven Parallel Manipulator (CDPM) with four governing geometric parameters, measured in millimetre (Fig. 1a):

• h — vertical distance between the base and platform anchor centers;
• D — shared diameter of the base and platform disks;
• r — radial distance of the lower-cable holder contact points from the joint center;
• a — spacing between successive lower-cable holder contact points.

#### Coordinate frames

Nine coordinate frames describe the system (Fig. 3): Frame 0 (global, fixed) and Frame 1 (body-fixed, rotating with joint angle θ) track the central joint rotation; Frames 2–3 mark the fixed base anchor boundaries; Frames 4–5 mark the moving platform anchors; Frames 6–8 describe the dynamic lower-cable holder geometry. The upper cable runs directly from Frame 2 to Frame 4. The lower cable runs from Frame 3, through the intermediate holder (Frame 6), to Frame 5, with the auxiliary segments (Frame 3→6 and Frame 8→5) staying constant in length and therefore cancelling out of the displacement calculation.

**Fig. 3:**
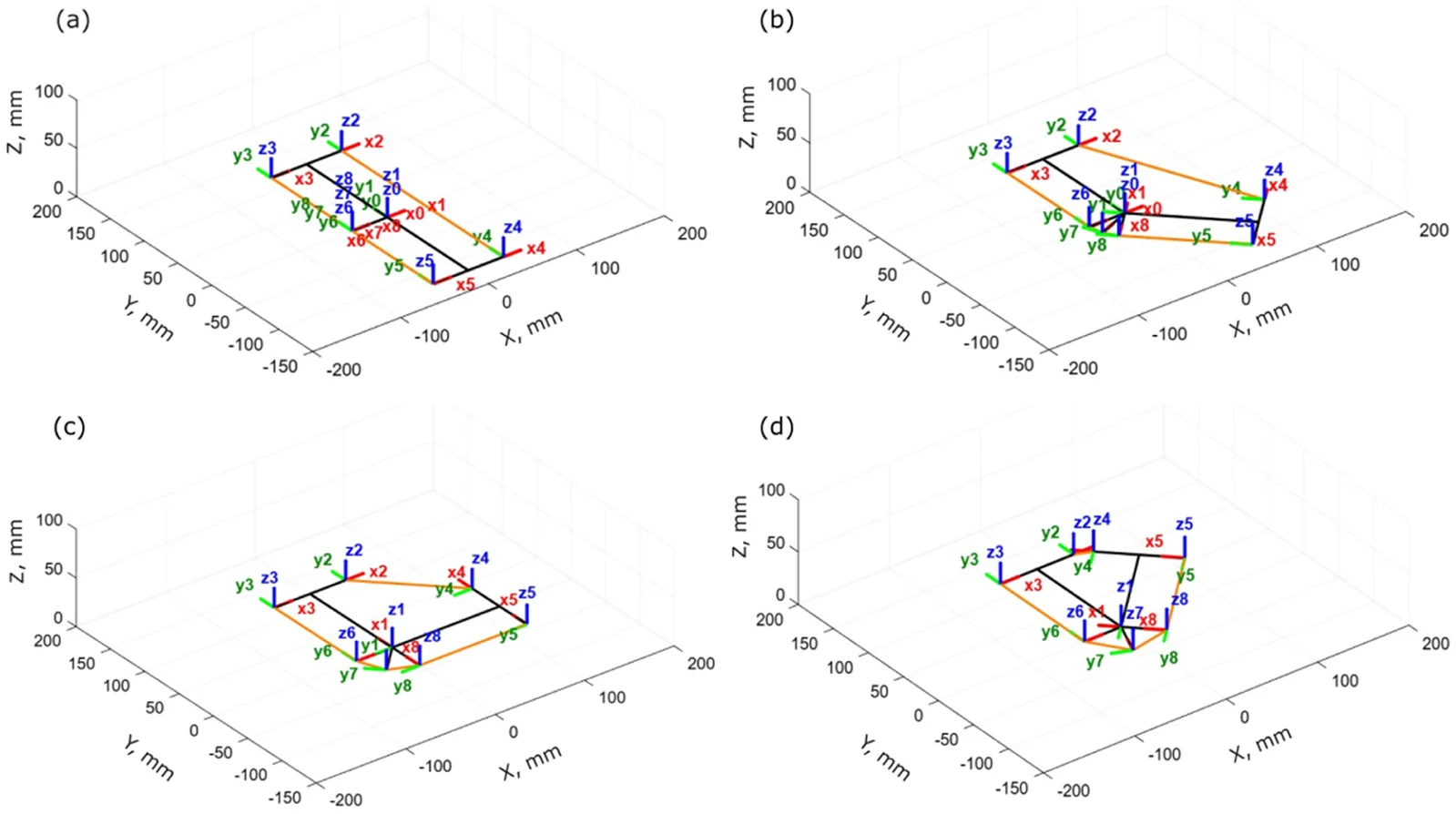
Coordinate frame representation of the elbow joint mechanism at successive rotational configurations: (a) Home position, (b) 45°, (c) 90°, and (d) 135° relative to the initial configuration.

#### Inverse kinematics

Solving the inverse kinematics problem allows for the determination of the displacement profiles for both the upper and lower actuator cables. The input parameter driving this geometric calculation is the central joint angle θ, which quantifies the angular rotation of the mobile platform relative to the static base. The transformation chain initiates by mapping the angular displacement of Frame 1 relative to the fixed global frame 0 via the homogeneous rotation matrix ${\;}^{0}A_{1}$, represented in Eq. (1):

(1) $${\;}^{0}A_{1}=[\begin{array}{cccc} cos\theta & -sin\theta & 0 & 0\\ sin\theta & cos\theta & 0 & 0\\ 0 & 0 & 1 & 0\\ 0 & 0 & 0 & 1 \end{array}],\;{\;}^{0}A_{2}=[\begin{array}{cccc} 1 & 0 & 0 & \frac{D}{2}\\ 0 & 1 & 0 & \frac{h}{2}\\ 0 & 0 & 1 & 0\\ 0 & 0 & 0 & 1 \end{array}],\;{\;}^{1}A_{4}=[\begin{array}{cccc} 1 & 0 & 0 & \frac{D}{2}\\ 0 & 1 & 0 & -\frac{h}{2}\\ 0 & 0 & 1 & 0\\ 0 & 0 & 0 & 1 \end{array}]$$

To calculate the spatial coordinates of the remaining functional frame locations, relative translation dimensions are incorporated into the global mapping matrix. The spatial path leading from the global home frame 0 to the upper base anchor at Frame 2 requires a translation of $\frac{D}{2}$ along the x-axis and a positive offset of $\frac{h}{2}$ along the y-axis, yielding the transformation matrix ${\;}^{0}A_{2}$. In a symmetrical fashion, reaching the platform anchor at Frame 4 from the rotating joint frame 1 requires a translation of $\frac{D}{2}$ along the relative x-axis and an offset of – $\frac{h}{2}$ along the relative y-axis, expressed through the matrix ${\;}^{1}A_{4}$ in Eq. (1).

Determining the position of the dynamic platform anchor at Frame 4 relative to the fixed global origin requires computing the composite kinematic chain. This is achieved by combining the initial joint rotation of angle θ with the relative structural translations along the local coordinate axes. Mathematically, this global position vector is evaluated through the direct matrix multiplication of ${\;}^{0}A_{1}$ and ${\;}^{1}A_{4}$, establishing the global transformation matrix ${\;}^{0}A_{4}$, as shown in Eq. (3):

(2) $${}^{0}A_{4}={}^{0}A_{1}\cdot {}^{1}A_{4}=\;[\begin{array}{cccc} cos\theta & -sin\theta & 0 & \frac{D}{2}cos\theta +\frac{h}{2}sin\theta \\ sin\theta & cos\theta & 0 & \frac{D}{2}sin\theta -\frac{h}{2}cos\theta \\ 0 & 0 & 1 & 0\\ 0 & 0 & 0 & 1 \end{array}]\;$$

#### Right-cable (cable 1) inverse kinematics

The absolute instantaneous length of the right cable $l_{1}\left(\theta \right)$ is determined by calculating the Euclidean distance between the fixed base anchor and the moving platform anchor in the global coordinate frame. Utilizing the coordinate mappings from the final column of the transformation matrices ${}^{0}A_{2}$ in Eq. (1) and ${}^{0}A_{4}$ in Eq. (2), the instantaneous cable length $l_{1}\left(\theta \right)$ and its corresponding displacement $\Delta l_{1}$ are formulated as shown in Eq. (3):

(3) $$l_{1}\left(\theta \right)=\frac{1}{\sqrt{2}}\sqrt{\left(h^{2}+D^{2}\right)+\left(h^{2}-D^{2}\right)\cos\theta -2hD\sin\theta },\;\text{and}\;\Delta l_{1}=l_{1}\left(\theta \right)-l_{1}\left(ref\right)$$

The term $l_{1}\left(ref\right)$ refers to the reference length of the right cable, which is equivalent to the distance between the base and platform in home position ($l_{1}\left(ref\right)$ = *h*), as shown in Fig. 1a.

#### Lower-cable (cable 2) inverse kinematics and collision avoidance

Managing interference between the central joint and the left cable requires a specialized routing architecture using four distinct orange anchor points, as shown in Fig. 1. The displacement between the first two points on the base and the last two points on the platform remains static during motion. Active displacement is modeled as two specific sections running between the base and platform contact points, with each section represented by the base of an isosceles triangle. As illustrated in Fig. 1b, two isosceles triangles are utilized, where each triangle's base corresponds to section a and its side corresponds to dimension r.

Because these paths are positioned close to the central axis, the cable is treated as a flexible bending element spanning three contact points (A, B, and C) to avoid collisions. Point A serves as the stationary home position on the base, Point B rotates by θ/2 through the central joint, and Point C rotates by the full joint angle θ. Positioned at a radius r from the joint center and separated by a uniform distance a, these points bound the total operational cable movement to 2a.

Based on Fig. 1b, the term a is mathematically modeled by Eq. (4) as half displacement of the second (lower) cable $\frac{\Delta l_{2}}{2}$ as a function of radius *r* and joint angle θ. Specifically, by bisecting the vertex angle of the isosceles triangle formed by the holder geometry into right triangles, the half-angle relationship yields a $\frac{\theta }{4}$ argument inside the sine function:

(4) $$\frac{a}{2}=r\cdot \sin\left(\frac{\theta }{4}\right),\;a=\frac{\Delta l_{2}}{2}=2r\cdot \sin\left(\frac{\theta }{4}\right),\;\;\Delta l_{2}=4r\cdot \sin\left(\frac{\theta }{4}\right),\;\;$$

To replicate the kinematic model for a new geometry:

1. Define h, D, r, and a for the selected design.
2. Assign coordinate frames following the scheme above (global frame, rotating body frame, base/platform anchors, holder frames).
3. Build the homogeneous transformation chain (Eq. (1)–(2)) to obtain anchor positions as a function of θ.
4. Compute the direct-path cable (Cable 1) length via the Euclidean-distance formula (Eq. (3)) and differentiate or tabulate over the target θ range.
5. For the left cable requiring holder-based routing, compute the holder-segment displacement (term a), and left cable displacement ($\Delta l_{2}$) via Eq. (4)

### Workspace sizing procedure (selecting h, and r)

The base-to-platform distance h was swept across four representative values relative to a fixed platform diameter D=80 mm: h=2D (160 mm), 3D (240 mm), 4D (320 mm), and 5D (400 mm) (Fig. 1b–d, Fig. 4). At h = 160 mm, structural collision between base and platform links halts rotation prematurely (≈126°, short of the 135° target). At h = 240 mm and above, the target 135° is reached without collision; larger h further increases clearance but linearly increases the required right cable stroke (Fig. 4a) and, as shown by the static analysis in Section 5 below (Fig. 5a), increases the peak cable tension.

**Fig. 4:**
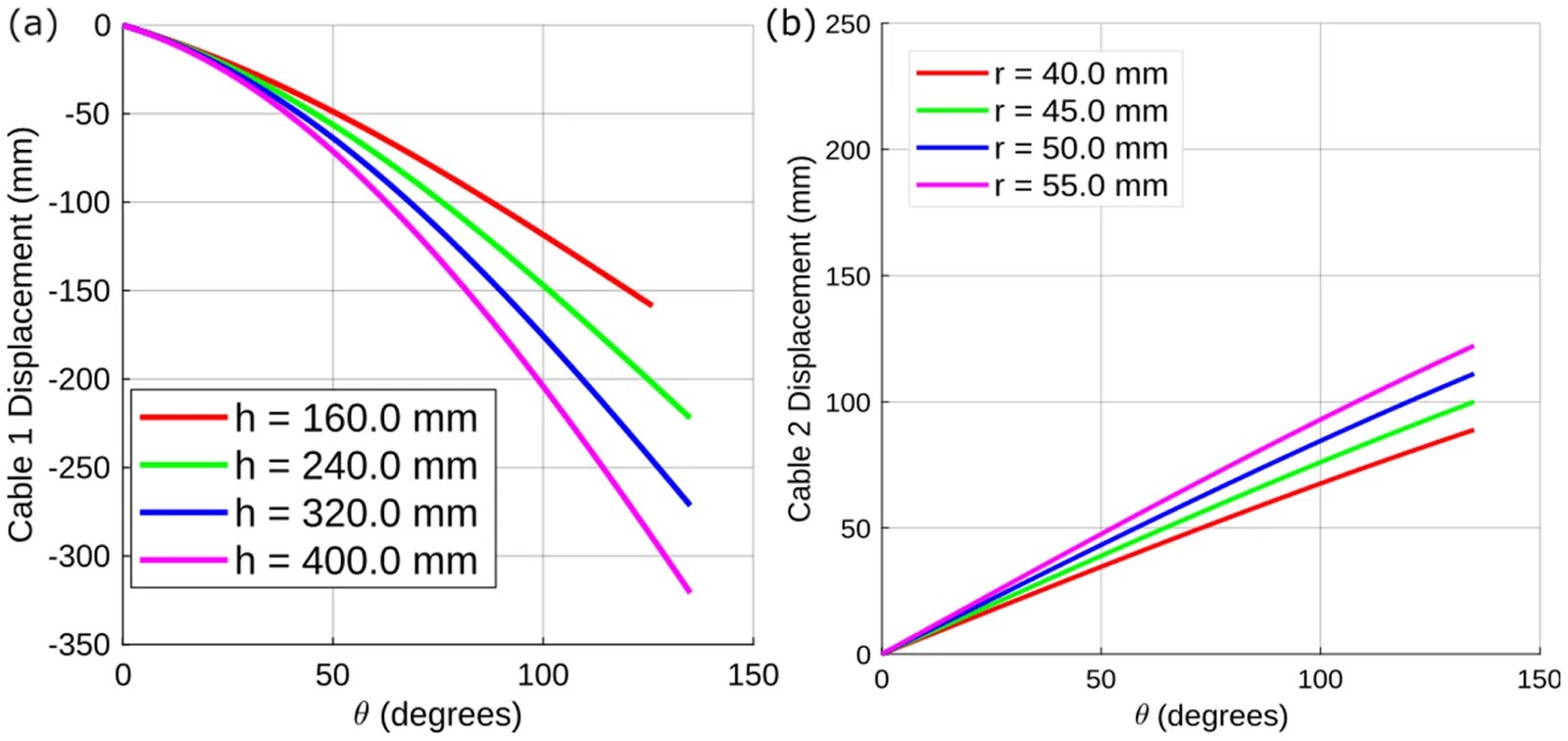
Cable displacement (∆l) characteristics with respect to elbow joint rotation (θ): (a) Right cable displacement for four values of the base-to-platform distance (h); (b) Variation of left cable displacement for four different cable holder radii (r).

**Fig. 5:**
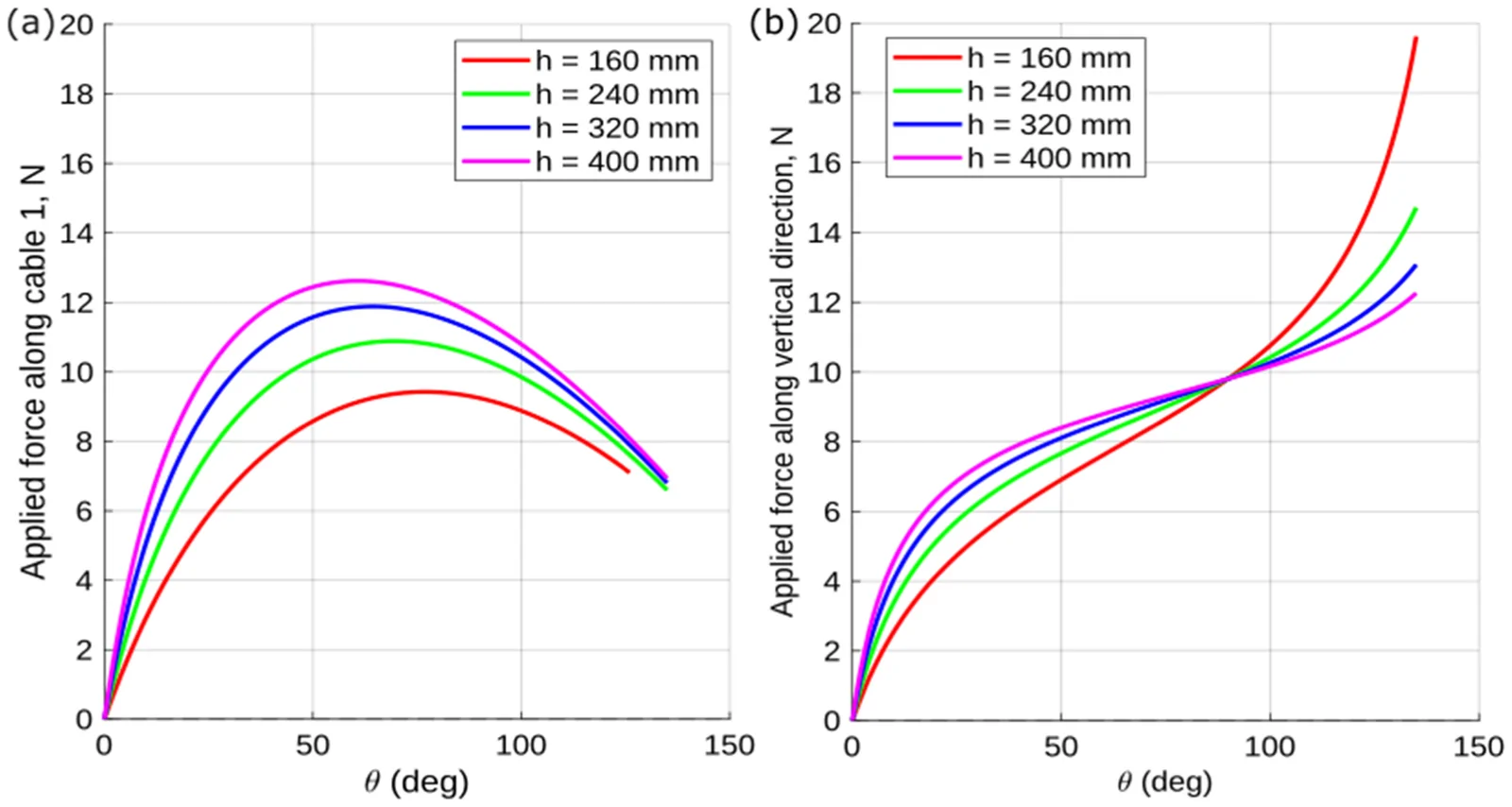
Analytical static force profiles vs. joint angle (θ). Graphs (a) and (b) show Cable 1 pulling force and vertical force across base-to-platform distances (h) when Cable 2 is fully extended.

This defines a three-step sizing procedure that other researchers can reuse for a different target ROM or spacing between the platform and base parts at a certain platform size:

**Step 1. Lower-bound h for collision-free rotation.** For the chosen platform diameter D and target maximum flexion angle θmax, increase h for the right cable until the simulated or CAD-checked rotation reaches θmax without base–platform interference. In this design, h = 3D (240 mm) was the minimum value for h clearing 135° interference-free.
**Step 2. Upper or lower bound h for force and stroke efficiency.** Since larger h values increase both the required right cable stroke (Fig. 4a) and peak actuation tension (Section 5), selecting the correct h in Step 1 is feasible to minimize motor winding length and actuator load. Consequently, the most preferable design out of four proposed parametric sweep points of h is achieved at h = 3D = 240 mm, which successfully satisfies the criteria for both steps 1 and 2. As detailed in Section 6, a cost function incorporating upper cable displacement and tension terms was employed to evaluate each scenario.
**Step 3. Select the holder radius** r **for the left cable via displacement sweep.** Repeat the parameter sweep across candidate radii using Fig. 4b to observe how the arc length around the joint impacts cable displacement. Because gravity assists the lower cable during pseudo-static operation, the holder radius is selected primarily to minimize total cable travel and prevent bulky spool enclosures. In this design, r=40mm was chosen to maintain a manageable stroke ($\Delta l_{2}\approx 88.6\;\text{mm}$) while ensuring sufficient clearance, with the cost-function weighting provided in Section 6.

### Static force modeling procedure

To characterize the cable-driven elbow joint system, we rigorously evaluate the actuation forces required to maintain static equilibrium. Static models are formulated to represent the forces acting vertically along the platform and right cable attachment points, as well as along the line of the right cable. In both models, second cable tension is not considered because platform assists to the left cable due to gravity. In the final model, the static force including the left cable line is characterized. Both linear and angular accelerations are neglected under a pseudo-static assumption due to low velocity of the actuators.

#### Idealized vertical-tension model

This model assumes that the upper cable tension vector remains purely vertical and parallel to the base link across all platform rotation angles. Balancing the cable's vertical upward force $F_{\text{ver}1}$ against the platform's gravitational load $F_{g}=mg$ (Fig. 1b) acting through its moment arm about the joint pivot ($M_{\text{ver}1}=M_{g}$) yields the closed-form tension approximation in Eq. (5):

(5) $$M_{ver1}=F_{ver1}\cdot \left(\frac{h}{2}sin\theta +\frac{D}{2}cos\theta \right),\;M_{g}=F_{g}\cdot \left(\frac{h}{2}sin\theta \right),\;F_{ver1}=\frac{mgh}{h+D\cdot \text{cot}\left(\theta \right)},\;0<\theta \leq 135^{\circ }\;$$

Fig. 5b illustrates the analytical static force profiles along the vertical direction ($F_{\text{ver}1}$) as a function of the elbow joint rotation angle θ for four different base-to-platform distances (h). As shown in the plot, all curves start near zero at the home position ($\theta =0^{\circ }$) and increase nonlinearly as the joint flexion angle grows. Near $90^{\circ }$, the curves intersect, after which their behaviors diverge significantly as the joint approaches its maximum flexion limit of $135^{\circ }$. Shorter links experience a sharp exponential rise in required vertical force in this upper range, with the h=160mm curve spiking close to 20 N, while longer links show a more gradual increase. This divergence at high angles reflects the mechanical constraints and moment arm changes depicted in Fig. 1b and d, where shorter base-to-platform distances face higher mechanical demands near full articulation.

#### Realistic single-cable line-of-action model

To account for the rotation-dependent geometry of the cable routing, the tension force vector $F_{1}$ from Fig. 1 is defined by pulling from the moving platform attachment point $p_{\text{platform}1}={\left[x_{\text{platform}1},y_{\text{platform}1}\right]}^{T}$ back toward the fixed base anchor $p_{\text{base}1}={\left[P_{\text{base}1x},P_{\text{base}1y}\right]}^{T}$ along the unit vector ${\overset{ˆ}{u}}_{1}$:

(6) $$F_{1}=F_{1}{\overset{ˆ}{u}}_{1}=\frac{F_{1}}{L_{1}\left(\theta \right)}\left(p_{\text{base}1}-p_{\text{platform}1}\right)=\frac{F_{1}}{L_{1}\left(\theta \right)}\;\left[\begin{array}{c} P_{\text{base}1x}-x_{\text{platform}1}\\ P_{\text{base}1y}-y_{\text{platform}1} \end{array}\right]$$

where $L_{1}\left(\theta \right)$ represents the instantaneous cable length computed from Eq. (3).

The restorative moment vector $M_{F1}$ produced about the central revolute joint origin (0,0) is obtained via the 3D vector cross-product of the position vector $p_{\text{platform}1}$ and the cable tension vector $F_{1}$:

(7) $$\;M_{F1}=p_{\text{platform}1}\times F_{1}=\left[\begin{array}{c} x_{\text{platform}1}\\ y_{\text{platform}1} \end{array}\right]\times \frac{F_{1}}{L_{1}\left(\theta \right)}\left[\begin{array}{c} P_{\text{base}1x}-x_{\text{platform}1}\\ P_{\text{base}1y}-y_{\text{platform}1} \end{array}\right]$$

Expanding the out-of-plane (z-component) determinant yields:

(8) $$M_{F1}=\frac{F_{1}}{L_{1}\left(\theta \right)}\left[x_{\text{platform}1}\left(P_{\text{base}1y}-y_{\text{platform}1}\right)-y_{\text{platform}1}\left(P_{\text{base}1x}-x_{\text{platform}1}\right)\right]$$

Canceling out the opposing cross-products ($x_{\text{platform}1}y_{\text{platform}1}$ terms) results in the definitive scalar moment equation expressed in Eq. (9), and the Cable 1 anchor point locations on the base and platform are specified:

(9) $$M_{F1}=\frac{F_{1}}{L_{1}\left(\theta \right)}\left(x_{\text{platform}1}P_{\text{base}1y}-y_{\text{platform}1}P_{\text{base}1x}\right),\;P_{base1}=\left[\begin{array}{c} \frac{D}{2}\\ \frac{h}{2} \end{array}\right],\;P_{platform1}=\left[\begin{array}{c} x_{platform1}\\ y_{platform1} \end{array}\right]=\left[\begin{array}{c} \frac{h}{2}sin\theta +\frac{D}{2}cos\theta \\ -\frac{h}{2}cos\theta +\frac{D}{2}sin\theta \end{array}\right]$$

Under pseudo-static motion, static equilibrium about the central joint pivot (0,0) requires the restorative moment generated by Cable 1 ($M_{F1}$) to balance the platform's gravitational moment ($M_{g}$):

(10) $$M_{F1}\left(\theta \right)=M_{g}\left(\theta \right)=\frac{mgh}{2}\sin\left(\theta \right)$$

By combining both Eqs. (3), (9), (10), we obtain the closed-form tension $F_{1}$ along the right cable (Eq. (11)), and the results of that tension at variable distance between base and platform h are plotted in Fig. 5a:

(11) $$\;F_{1}=\frac{mgh\text{sin}\theta \cdot \sqrt{\left(h^{2}+D^{2}\right)+\left(h^{2}-D^{2}\right)\text{cos}\theta -2hD\text{sin}\theta }}{\sqrt{2}\left(hD\text{cos}\theta +\frac{D^{2}-h^{2}}{2}\text{sin}\theta \right)}\;$$

#### Antagonistic two-cable model

To formulate the complete static equilibrium model, we establish explicit sign conventions for all joint moments: counter-clockwise (CCW) moments about the joint pivot [0,0] are defined as positive, while clockwise (CW) moments are negative. Under this convention, Cable 1 exerts a positive (CCW) moment $M_{F1}>0$ pulling the arm toward flexion, whereas Cable 2 exerts a negative (CW) moment $M_{F2}<0$ pulling toward extension, and the gravitational load creates a negative (CW) moment $M_{g}<0$.

The net restorative moment $M_{\text{pivot}}$ about the joint center must satisfy static equilibrium, as shown in Eq. (12):

(12) $$\sum M_{pivot}=M_{F1}-M_{F2}-M_{g}=0,\;M_{F2}=\;F_{2}\cdot r_{2\left(\theta \right)},\;r_{2\left(\theta \right)}=\frac{\Delta l_{2}}{d\theta }=\frac{d}{d\theta }\left[4r\cdot \sin\left(\frac{\theta }{4}\right)\right]=r\cdot \cos\left(\frac{\theta }{4}\right)\;$$

Using virtual work principles, the effective moment arm $r_{2}\left(\theta \right)$ of Cable 2 is related to its kinematic displacement derivative by $r_{2}\left(\theta \right)=\frac{d\Delta l_{2}\left(\theta \right)}{d\theta }$, where the joint angle θ is expressed in radians for dimensional consistency (yielding mm/rad). Differentiating Eq. (4) with respect to θ yields $r_{2(\theta )}$ in Eq. (12).

Substituting $M_{F1}\left(\theta \right)$ from Eq. (9), $M_{F2}\left(\theta \right)=F_{2}\cdot r_{2}\left(\theta \right)$ from Eq. (12), and the gravitational moment $M_{g}\left(\theta \right)$ from Eq. (10) into the moment balance equation ($\sum M_{\text{pivot}}=0$), and solving explicitly for $F_{1}$, yields Eq. (13):

(13) $$F_{1}=\frac{\left[mgh\cdot \sin\left(\theta \right)+2F_{2}\cdot r\cos\left(\frac{\theta }{4}\right)\right]\cdot \sqrt{\left(h^{2}+D^{2}\right)+\left(h^{2}-D^{2}\right)\cos\left(\theta \right)-2hD\sin\left(\theta \right)}}{\sqrt{2}hD\cos\left(\theta \right)+\left(\frac{h^{2}-D^{2}}{\sqrt{2}}\right)\sin\left(\theta \right)}$$

Reduction to Single-Cable Case: Setting $F_{2}=0\;N$ (releasing the secondary cable tension) eliminates the secondary moment term, reducing the numerator directly to $mgh\cdot \sin\left(\theta \right)\cdot L_{1}\left(\theta \right)$, which simplifies identically to the single-cable equilibrium expression in Eq. (11).

To replicate the static analysis for a new geometry:

1. Fix the platform mass *m* and use *g* = 9.81 m/s².
2. Compute the tension F_ver1_ (Eq. (5)) along the vertical direction as a first-pass sanity check across the target θ range.
3. Compute the realistic single-cable tension *F₁* (Eq. (11)) using the actual anchor coordinates (Eq. (9)) and cable-length expression (Eq. (3)).
4. If an antagonistic second cable is present, derive its effective moment arm from its own displacement function by differentiating *Δl₂*(θ), and substitute into the combined moment balance (Eq. (12)) to obtain F₁ as a function of the chosen left-cable pre-tension F₂.
5. Sweep *h* (or other free geometric parameters) through the candidate values from Section 4 to compare peak tension and select the configuration minimizing actuator load while satisfying the collision-free constraint.

At $\theta =90^{\circ }$, gravity's interaction with the antagonistic layout creates distinct mechanical demands for each cable. Because Cable 2 is routed at the bottom, the downward weight of the platform aligns with its pulling direction, allowing gravity to assist Cable 2 and reduce its required actuation effort. Conversely, top-mounted Cable 1 must pull upward to lift the platform against gravity, bearing its full structural weight alongside the pre-tensioned antagonistic load.

### Geometric parameter selection procedure

To formally justify the selection of the core structural parameters—the base-to-platform distance h and the lower cable holder radius r—a unified multi-objective evaluation framework was established. The kinematic derivations in Section 3 and the static force formulations in Section 5 reveal conflicting design trade-offs: increasing h improves Cable 1 leverage but increases stroke, while increasing r alters Cable 2 travel requirements. To resolve these trade-offs prior to hardware fabrication, objective cost functions were formulated.

For Cable 1, the cost function $J_{1}\left(h\right)$ incorporates both peak static tension and maximum cable displacement, as shown in Eq. (14):

(14) $$J_{1}\left(h\right)=w_{F}\cdot \frac{F_{1,\text{max}}\left(h\right)}{F_{1,\text{norm}}}+w_{\Delta l}\cdot \frac{\left|\Delta l_{1,\text{max}}\left(h\right)\right|}{\Delta l_{1,\text{norm}}},\;J_{2}\left(r\right)=\frac{\left|\Delta l_{2,\text{max}}\left(r\right)\right|}{\Delta l_{2,\text{norm}}}$$

where $F_{1,\text{max}}\left(h\right)=\max\left(F_{1}\left(\theta ,h\right))\right)$ and $|\Delta l_{1,\text{max}}\left(h\right)|$ denote the peak static tension and maximum required cable displacement, respectively. The normalization factors are $F_{1,\text{norm}}=12.6\;N$ and $\Delta l_{1,\text{norm}}=320\;\text{mm}$. The weighting coefficients are set to $w_{F}=0.5$ and $w_{\Delta l}=0.5$ as equal weighting coefficients to establish a balanced baseline, penalizing excessive actuator tension load and large cable spool travel equally, as minimizing motor mechanical stress and avoiding bulky housing or spool constraints are treated as equally vital priorities for a compact cable-driven elbow joint.

Conversely, because gravity assists the lower cable (Cable 2) during pseudo-static operation, its static payload resistance is negligible in the open-loop static model ($F_{2,\text{max}}\approx 0$). Consequently, the cost function for the lower cable holder radius, $J_{2}\left(r\right)$, is formulated exclusively around minimizing total cable stroke to prevent bulky spool enclosures in Eq. (14), where $|\Delta l_{2,\text{max}}\left(r\right)|$ represents the maximum required displacement of Cable 2 evaluated from Eq. (4), and $\Delta l_{2,\text{norm}}=121.82\;\text{mm}$ is the upper-bound normalization factor across the radius domain r∈[40,55]mm. While Cable 2 is sized purely based on stroke minimization and geometric clearance (r≥D/2) in this geometric prototyping stage, active pre-tensioning and dynamic force variations are subsequently managed during closed-loop hardware-in-the-loop execution via the controller (Section 7).

#### Base-to-platform distance selection (h parameter for cable 1)

Table 1 summarizes the parametric sweep and cost evaluation for the base-to-platform distance h at a fixed platform diameter D=80mm.

**Table 1:** Parametric sweep and weight sensitivity analysis for base-to-platform distance (h) at a fixed joint diameter D=80mm using varying cost function weights ($W_{1},W_{2}$).

Base-to-platform distance (h)	Highest Displacement of Cable 1 (Δl1,max​)	Peak Tension of Cable 1 (F1,max​)	Cost Function J1​(h)			Design Trade-off Verdict
			W1=0.5 W2=0.5	W1=0.75 W2=0.25	W1=0.25 W2=0.75	
**160** **mm** (2*D*)	158 mm	9.4 N	Infeasible (∞)			Rejected: Limited range of motion ($126^{\circ }$)
**240** **mm** (3*D*)	221 mm	10.9 N	0.78 (Minimum)	0.73 (Minimum)	0.82 (Minimum)	Selected: Preferred candidate balancing force and stroke.
**320** **mm** (4*D*)	270 mm	11.9 N	0.89	0.86	0.9	Imperfect: Unnecessary spool capacity penalty.
**400** **mm** (5*D*)	>320 mm	12.6 N	1.00	1.00	1.00	Rejected: Maximum cost penalty across both metrics.

As detailed in Table 1, h=160mm (2D) yields lower absolute force and displacement values, but fails the feasibility constraint due to mechanical binding at $126^{\circ }$. Among all viable candidates, h=240mm (3D) achieves the lowest-cost feasible candidate within the tested set ($J_{1}=0.78$), establishing it as the most preferable structural length for Cable 1.

#### Holder radius selection (r parameter for cable 2)

Table 2 outlines the parametric evaluation for the lower cable holder radius r evaluated across the range of 40mm to 55mm at a fixed base-to-platform distance h=240mm and platform diameter D=80mm. Under pseudo-static gravity assistance, Cable 2 experiences negligible static payload resistance in the open-loop static model, meaning its holder radius selection is governed primarily by minimizing total cable stroke and satisfying geometric clearance boundaries rather than balancing static compressive loads. Consequently, the cost function $J_{2}\left(r\right)$ evaluates exclusively normalized cable displacement, as shown in Eq. (14).

**Table 2:** Parametric sweep for holder radius (r) based on displacement minimization.

Holder Radius (r)	Highest Displacement of Cable 2 (Δl2,max​)	Cost Function J2​(r)	Design Trade-off Verdict
40mm	≈88.6mm	0.73**(Minimum)**	**Selected:** Satisfies r≥D/2 clearance limit with minimal stroke
45mm	≈99.7mm	0.82	**Imperfect:** Increased cable travel penalty
50mm	≈110.8mm	0.91	**Imperfect:** Higher spool capacity required
55mm	≈121.82mm	1.0	**Rejected:** Maximum stroke penalty and increased joint bulk

As detailed in Table 2, evaluating the holder radius across the valid geometric domain (r≥40mm) demonstrates that r=40mm is the preferable candidate, satisfying the critical clearance threshold required to prevent physical wall collisions while achieving the lowest cost function score ($J_{2}=0.73$) with a minimized cable stroke ($\Delta l_{2,\text{max}}\approx 88.6\;\text{mm}$). Conversely, larger radii such as r=50mm or 55mm unnecessarily inflate cable travel ($\Delta l_{2,\text{max}}\geq 110\;\text{mm}$) and spool bulk. Active pre-tensioning and dynamic force behavior for Cable 2 are subsequently managed during closed-loop hardware-in-the-loop operation rather than open-loop geometric sizing.

Varying the weighting coefficients ($W_{1},W_{2}\in \left[0.25,0.75\right]$) demonstrates that h=240mm maintains the minimum cost score $J_{1}\left(h\right)$ compared to other candidate geometries across both force-dominant and stroke-dominant preferences, as demonstrated in Table 1. Consequently, the selection of a 240mm base-to-platform distance still remains preferable despite variations in design priorities.

Consequently, the cost function for the lower cable holder radius, $J_{2}\left(r\right)$, is formulated exclusively around minimizing total cable stroke to prevent bulky spool enclosures as shown in Eq. (14), where $|\Delta l_{2,\text{max}}\left(r\right)|$ represents the maximum required displacement of Cable 2 evaluated from Eq. (4), and $\Delta l_{2,\text{norm}}=121.82\;\text{mm}$ is the upper-bound normalization factor across the radius domain r∈[40,55]mm.

### Hardware-in-the-loop experimental setup

The prototype was evaluated using a hardware-in-the-loop (HIL) architecture (Fig. 6, Fig. 7) comprising a host PC running MATLAB/Simulink interfaced through a National Instruments (NI) SCB-68A DAQ board, a regulated benchtop DC power supply, two rotational EC motors driven by Maxon ESCON 50/5 motor controllers, an AMT 102-V incremental encoder on the joint axis, and two miniature S-type force sensors routed through a GSV-1A4 amplifier.

**Fig. 6:**
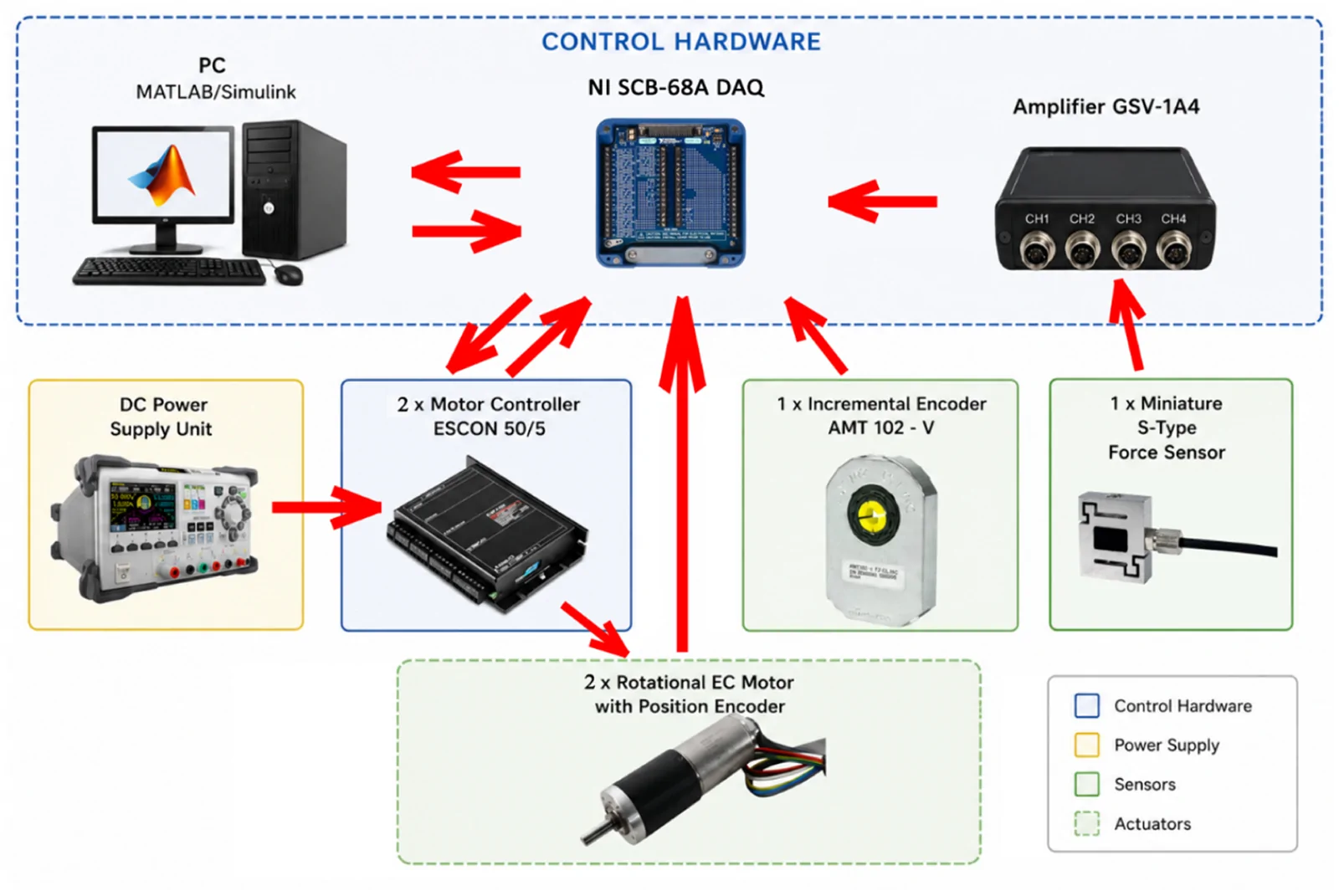
Hardware architecture of the integrated mechatronic control system for the compact elbow joint, highlighting the position and cable-tension control framework together with the power supply, control hardware, actuators, and sensors.

**Fig. 7:**
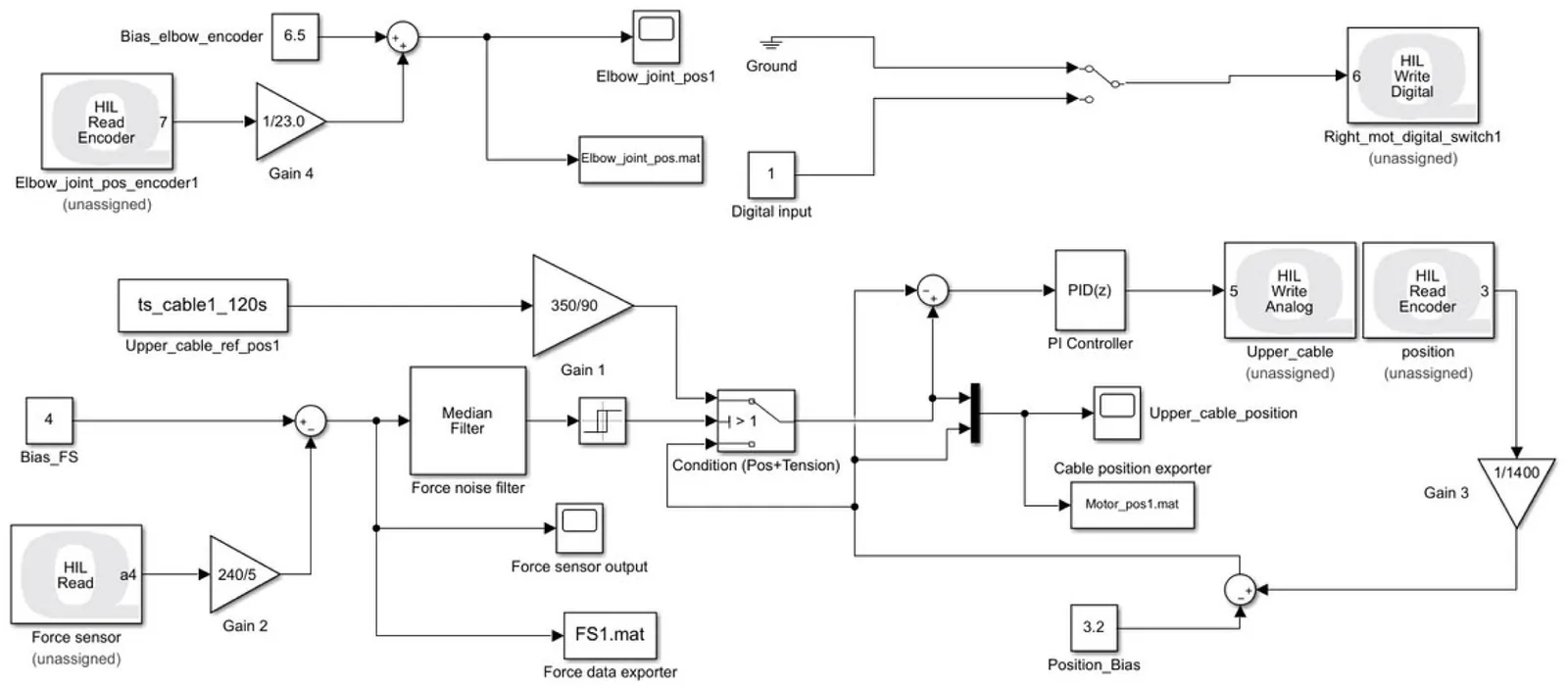
MATLAB/Simulink implementation of the hardware-in-the-loop (HIL) control system for the proposed elbow joint mechanism. The controller integrates dynamic switching between position control and force-threshold regulation to maintain positive cable tension and suppress slack during real-time trajectory tracking.

The closed-loop HIL control strategy combines inverse-kinematic trajectory generation with real-time force-threshold monitoring to ensure position tracking while maintaining positive cable tension. The control software runs two symmetric control channels in Simulink (one per cable) that incorporate a digital safety interlock for rapid actuator isolation alongside an encoder processing block converting raw quadrature counts into real-time joint angle feedback. Actuation is delivered by the two Maxon ESCON 50/5 motor controllers operating in current-control mode, interfaced via the NI SCB-68A DAQ board to the PC. The mechanism employs high-stiffness 2.5−mm steel actuation cables. Cable tension signals are continuously captured via the miniature inline load cells, amplified using the GSV-1A4 signal conditioner, and pre-filtered in Simulink using a median filter to suppress sensor noise.

To handle noise spikes and mitigate chattering when switching between position tracking and force-threshold regulation, a dedicated signal conditioning block is integrated directly into the real-time control pipeline. High-frequency sensor noise and transient mechanical impacts on the inline load cell can cause raw force measurements to momentarily cross the regulation threshold, leading to rapid toggling at the conditional switch and causing chattering in the motor voltage signal. To suppress impulse measurement noise, the raw force sensor output is processed using a median filter, introducing a bounded group delay (0.25s at 20Hz) that remains suitable for pseudo-static movement. The median filter effectively removes amplitude spikes and high-frequency load cell noise, substantially reducing transient toggling at the conditional switch and helping stabilize setpoint selection for the downstream discrete proportional-integral controller, preserving smooth voltage outputs to the motor drivers and maintaining positive cable tension throughout the movement.

Real-time joint tracking and tension regulation are executed via discrete proportional-integral (PI) controllers assigned to each cable loop, tuned to $K_{p}=40$ and $K_{i}=1$. As formalized in Algorithm 1, system control priority is explicitly granted to dynamic tension regulation and structural safety over nominal setpoint tracking. Under standard operating conditions, the primary cable (Cable 1) executes inverse-kinematic setpoints to control the platform rotation, while the antagonistic cable (Cable 2) provides continuous cable tension that yields platform position stabilization. To prevent cable derailment, control chattering, or backlash, the loop enforces a dual-threshold hysteresis slack prevention condition: if the measured tension $F_{i}$ on either cable drops below $F_{\text{min}}=1.0\;N$, Algorithm 1 overrides nominal kinematic tracking to apply an online length correction step, shortening the setpoint by q ($l_{i}\leftarrow l_{i}-q$) until tension recovers past the upper release threshold $F_{\text{upper}}=1.2\;N$. In the event that measured tensions for both cables fall below $F_{\text{min}}$ simultaneously, priority is assigned to Cable 1 to maintain joint angle stability while Cable 2 holds its nominal path, preventing conflicting actuator commands and excessive antagonistic co-contraction. Maximum current and torque thresholds are handled separately at the motor driver level to ensure structural safety, allowing the algorithm to focus exclusively on maintaining positive tension stability across the complete 135° range of motion.

**Algorithm 1:** Joint angle tracking with antagonistic tension regulation.

1: **Algorithm 1: Joint Angle Tracking with Antagonistic Tension Regulation**. 2: **Input:** Initial cable lengths $l_{i}\left(0\right)$, sensor-measured cable forces $F_{i}\left(k\right)$ (i∈{1,2}), minimum allowable tension threshold $F_{i,\text{min}}=1.0\;N$ (lower threshold), upper release threshold $F_{i,\text{upper}}=1.2\;N$, maximum allowable tension threshold $F_{i,\text{max}}$, nominal joint trajectory $\theta_{\text{ref}}\left(k\right)$, length correction factor q=0.5mm, sampling period T_s_ = 0.05 s (20 Hz), median filter window *W* = 5 3: **Output:** Reference actuator position $\varphi_{\text{ref},\;i}\left(k\right)$, (i=1,2) or Safety Shutdown Command 4: **Initialize:**$\text{slack}\_\text{fla}g_{1}\leftarrow \text{FALSE}$, $\text{slack}\_\text{fla}g_{2}\leftarrow \text{FALSE}$ 5: for discrete time step k = 1, 2, 3, … do 6: Acquire joint feedback θ(k) and measured cable forces $F_{i,\;raw}\left(k\right)$ for i∈{1,2}; 7: $\;F_{i}\left(k\right)$ ← MedianFilter ($F_{i,\;raw}\left(k\right),\;W$); /* Filter force measurements */ 8: /* Check safety over-tension condition */ 9: for cables i ← 1 to 2 do 10: if $F_{i}\left(k\right)>F_{i,\text{max}}$ then Trigger Safety Over-Tension Shutdown; Zero motor current commands (*I_cmd_* = 0); Exit / Halt System; 11: end if 12: end for 13: /* Step 1: Kinematic Setpoint Evaluation */ 14: Compute nominal cable lengths via inverse kinematics: 15: $l_{i}\left(k\right)\leftarrow \text{Compute}\;\text{Inverse}\;\text{Kinematics}\left(\theta_{\text{ref}}\left(k\right)\right)$; 17: /* Step 2: Hysteresis State Evaluation */ 18: for cables i ← 1 to 2 do 19 if $F_{i}\left(k\right)<F_{i,\text{min}}$ then 20: slack_flag_i ← TRUE; /* Activate slack compensation */ 21: else if $F_{i}\left(k\right)>F_{i,\text{upper}}$ then 22: slack_flag_i ← FALSE; /* Deactivate compensation, return to tracking */ 23: end if 24: end for 25: /* Step 3: Antagonistic Coordination Logic */ 26: if slack_flag_1 == TRUE and slack_flag_2 == TRUE then 27: /* Master–Slave Priority Rule for simultaneous low tension */ 28: $l_{1}\left(k\right)$ ← $l_{1}\left(k\right)$- q; /* Primary cable (Cable 1) priority for joint angle */ 29: $\;l_{2}\left(k\right)$ ←$l_{2}\left(k\right)$; /* Secondary cable (Cable 2) holds path */ 30: else if slack_flag_1 == TRUE then 31: $l_{1}\left(k\right)$ ← $l_{1}\left(k\right)$- q; 32: else if slack_flag_2 == TRUE then 33: $l_{2}\left(k\right)$← $l_{2}\left(k\right)$ - q; 34: end if 35: /* Step 4: Actuator Command Mapping */ 36: for cables i ← 1 to 2 do 37: Map cable lengths $l_{i}\left(k\right)$ to motor commands with gear ratio N = 120: 38: $\;\varphi_{\text{ref},\;i}\left(k\right)$*← (*$l_{i}\left(k\right)$*/ r*_spool_) * (180 / π) * N; 39: end for 40: Send updated reference actuator position commands $\varphi_{\text{ref},\;1}\left(k\right)$, $\varphi_{\text{ref},\;2}\left(k\right)$ to motor controllers; 41: Delay until next execution period T_s_; 42: end for

In practice, Algorithm 1 actively enforces a constant minimum bias pretension $F_{\text{preload}}$ (where $F_{2,\text{min}}>1\;N$) on Cable 2 across the entire range of motion to maintain structural tautness, prevent slack, and ensure continuous antagonistic stiffness. Under this real-time pretension strategy, the control loop maintains Cable 2 above a minimum pre-tension threshold ($F_{2}\geq F_{\text{preload}}$), allowing the actual force to vary dynamically with the joint angle during antagonistic operation.

Cable 1 then dynamically counteracts both the gravitational moment and this internal antagonist pretension, ensuring positive tension is guaranteed in both cables over the full range of motion.

To ensure reproducibility and specify the control loop, the hardware parameters, sensing specifications, and conversion mappings represented in Table 3 are detailed as follows. The empirical tension correction increment is set to q=0.5mm, selected based on offline step-response tuning to achieve rapid tension recovery. Raw cable-tension readings from the inline load cell are pre-filtered using a median filter (W=5 samples, corresponding to a 0.25s group delay at 20Hz) to reject isolated measurement impulse noise prior to threshold evaluation. To prevent control chattering around the force regulation boundary caused by sensor noise or load fluctuations, an explicit hysteresis band was integrated into the conditional switching logic (Fig. 7 and algorithm 1). The state transition is governed by a relay mechanism with a lower threshold of $F_{\text{lower}}=F_{\text{min}}=1.0\;N$ (triggering slack compensation) and an upper threshold of $F_{\text{upper}}=1.2\;N$ (returning to nominal trajectory tracking). The force sensor features an operational range of 0−100N ($F_{\text{range}}$), a resolution of $F_{\text{res}}=0.05\;N$, and a full-scale calibration error of $e_{\text{cal}}\leq \pm 0.2\%$.

**Table 3:** Controller, hardware, and sensing parameters for the elbow joint with an antagonistic cable pair.

Category	Parameter	Symbol	Value	Unit
**Control and Sampling**	Sampling period	$T_{s}$	0.05 (20 Hz)	S
	Proportional gain	$K_{p}$	40	—
	Integral gain	$K_{i}$	1	$s^{-1}$
	Reference motor position	$\phi_{\text{ref},i}\left(k\right)$	$\left(\frac{l_{i}\left(k\right)}{r_{\text{spool}}}\right)\cdot \left(\frac{180}{\pi }\right)\cdot N$	deg
	Measured motor position	$\phi_{i}\left(k\right)$	From encoder feedback	deg
	Motor position tracking error	$e_{\phi ,i}\left(k\right)$	$\phi_{\text{ref},i}\left(k\right)-\phi_{i}\left(k\right)$	deg
	Corrective motor position command	$\phi_{\text{PI},i}\left(k\right)$	$K_{p}e_{\phi ,i}\left(k\right)+K_{i}T_{s}\sum_{j=0}^{k}e_{\phi ,i}\left(j\right)$	deg
	Tension correction increment	q	0.5	mm
	Minimum tension threshold (lower trigger)	$F_{\text{min}}$ (or $F_{\text{lower}}$)	1.0	N
	Upper release	$F_{\text{upper}}$	1.2	N
**Hardware and Spool**	Motor-spool radius	$r_{\text{spool}}$	15.0	mm
	Motor gear ratio	N	120:1	—
	Operating voltage	$V_{\text{op}}$	24.0	VDC
	Peak operational current limit	$I_{\text{limit}}$	2.5	A
	Software motor torque limit	$\tau_{\text{limit}}$	0.21 (motor) / 25.2 (spool)	N·m
**Sensing and Filtering**	Force sensor measurement range	$F_{\text{range}}$	0−100	N
	Force sensor resolution	$F_{\text{res}}$	0.05	N
	Force sensor calibration error	$e_{\text{cal}}$	≤±0.2	%
	Quadrature encoder resolution	$E_{\text{res}}$	4096	counts/rev
	Median filter window length	W	5 (250 ms)	samples

For kinematic and actuation mapping, motor position is tracked using a quadrature encoder with

$E_{\text{res}}=\;4096\;\text{counts}/\text{revolution}$ connected through a 120:1 planetary gearbox to a motor spool with radius $r_{\text{spool}}=15.0\;\text{mm}$. The output variable of the tension PI controller is defined as the corrective motor angular position $\theta_{\text{PI}}$ (deg).

Real-time joint tracking and tension regulation are executed through discrete proportional-integral (PI) controllers assigned to each cable loop, tuned to $K_{p}=40$ and $K_{i}=1$. As formalized in Algorithm 1, system control priority is explicitly granted to dynamic tension regulation and structural safety over nominal setpoint tracking.

The primary cable (Cable 1) control loop processes a position setpoint $p_{\text{cmd}}$ that is dynamically assigned by the conditional switching logic. Under standard operating conditions, $p_{\text{cmd}}$ equals the nominal inverse-kinematic trajectory. However, if the measured tension $F_{i}$ falls below the minimum threshold $F_{\text{min}}=1\;N$, the loop triggers a tension recovery override, adjusting the position command to re-establish required cable tautness.

The discrete PI controller block acts directly on the motor shaft position tracking error $e_{\phi ,i}\left(k\right)=\phi_{\text{ref},i}\left(k\right)-\phi_{i}\left(k\right)$, where $\phi_{\text{ref},i}\left(k\right)$ is the reference motor position derived from the cable length $l_{i}\left(k\right)$ (Algorithm 1) and $\phi_{i}\left(k\right)$ is the actual motor position measured by the actuator encoder. Note that ϕ denotes motor rotation, distinguishing it from the central elbow joint angle θ. The resulting corrective motor angular command $\phi_{\text{PI},i}\left(k\right)$ generated by the controller is expressed in Eq. (15):

(15) $$\phi_{\text{PI},i}\left(k\right)=K_{p}e_{\phi ,i}\left(k\right)+K_{i}T_{s}\sum_{j=0}^{k}e_{\phi ,i}\left(j\right)$$

where $T_{s}=0.05\;s$ (20Hz) is the sampling period. The output command $\phi_{\text{PI},i}$ is sent directly to the Maxon ESCON 50/5 motor driver operating in current-control mode to regulate the motor current command $I_{\text{cmd}}$.

To prevent actuator damage and preserve cable integrity, the system operates under strict operational limits rather than relying solely on the driver's maximum hardware ratings: an operating voltage of

$V_{\text{op}}=24.0\;V\;\text{DC}$ (allowable range of motor driver voltage: 12−48V), a peak operational current limit of $I_{limit}=1.5\;A$ (driver allowable: 10.0A), and a software-enforced motor torque limit of

$\tau_{\text{limit}}=0.21\;N\cdot m$ (yielding a gear-reduced continuous output torque capacity up to 25.2N·m at the spool). Finally, if the measured tension $F_{i}$ exceeds the maximum safety threshold $F_{\text{max}}$, the process immediately shuts down by triggering an automatic safety halt, zeroing the motor current command ($I_{\text{cmd}}=0\;A$), and flagging an over-tension fault error.

Calibration and signal conditioning:

1) Calibrate each S-type force sensor against known loads to obtain a load-to-voltage mapping.
2) Apply a median filter in MATLAB to the raw force signal to suppress high-frequency.
3) Run the control loop at a fixed sample time of 0.05 s.
4) Minimize and insulate all electrical wiring to reduce the noise, short circuit, and environmental interference.

To replicate the HIL setup with different hardware, match the four functional blocks (control/DAQ, power, actuation, sensing) and preserve the same control logic: independent symmetric loops per cable, a force threshold to switch between position and force regulation, and a median filter on the force signal tuned to the sensor noise floor and the required loop responsiveness.

## Method validation

To evaluate the validity and accuracy of the established mechanism, experimental validation was conducted on the antagonistic cable-driven elbow testbed across the joint’s operational range of motion. Two complementary validation procedures were conducted on the same 3D-printed prototype (h = 240 mm, D = 80 mm, r = 40 mm): (1) kinematic validation and (2) static-force validation.

### Kinematic validation

The theoretical cable displacement profiles obtained from the inverse kinematic model were compared with the corresponding experimental measurements for both right and left cables. Overall, the experimental results follow the predicted trajectories throughout the elbow joint range of motion, demonstrating the validity of the proposed kinematic formulation. For the right cable (Cable 1) in Fig. 9a, the measured displacement almost approaches the result of the theoretical model, with only a slight increase in deviation at larger flexion angles. Similarly, the experimental displacement of left cable (Cable 2) in Fig. 9b matches the analytical prediction, although a slightly larger discrepancy is observed during the initial stage of the motion before the two curves converge at higher joint angles.

The experimental trajectories were generated by commanding the motors using the cable displacement values computed from the theoretical inverse kinematics (IK) formulation. In other words, the required motor positions were determined directly from the analytical IK solution, and the measured cable displacements were subsequently compared with these theoretical predictions. Consequently, any difference between the two curves primarily reflects the physical characteristics of the experimental prototype rather than inaccuracies in the kinematic model itself.

Specifically, the experimental cable displacements ($\Delta l_{\text{exp},\;i}$) were derived from the motor shaft rotation angles recorded by the incremental encoders, translated into linear travel using the effective spool radius ($r_{\text{spool}}$), the spool circumference ($2\pi r_{\text{spool}}$), and the motor transmission gear ratio (N) via the relation (Eq. (16)):

(16) $$\Delta l_{\text{exp},\;i}=\left(\frac{\phi_{i}}{360^{\circ }}\right)\cdot 2\pi r_{\text{spool}}\cdot \frac{1}{N},\;i=1,2$$

This mapping converts the rotational encoder counts ($\phi_{i}$) into linear cable displacement, allowing a direct, point-by-point comparison against the theoretical inverse kinematics predictions.

Kinematic tracking performance was evaluated over N=5 repeated trials using a data acquisition sampling frequency of $f_{s}=20.0\;\text{Hz}$ ($T_{s}=0.05\;s$), yielding $N_{p}=2,401$ data points per trial. Throughout the entire range of motion ($0^{\circ }$ to $135^{\circ }$), measured linear cable speed spanned between 0.08mm/s and 4.76mm/s (Fig. 8c,d), alongside an average central joint angular velocity of approximately $\dot{\theta }=1.125^{\circ }/s$. Throughout the test, maximum travel spanned 240mm for the primary cable and 88.6mm for the secondary cable across a total span of 120s. Consequently, Cable 1 achieved an average speed of 2.0mm/s with a mean acceleration of $0.033\;\text{mm}/s^{2}$, while the corresponding results for Cable 2 were roughly three times lower. These low velocities and accelerations support the use of a pseudo-static approximation for the present benchtop experiments.

**Fig. 8:**
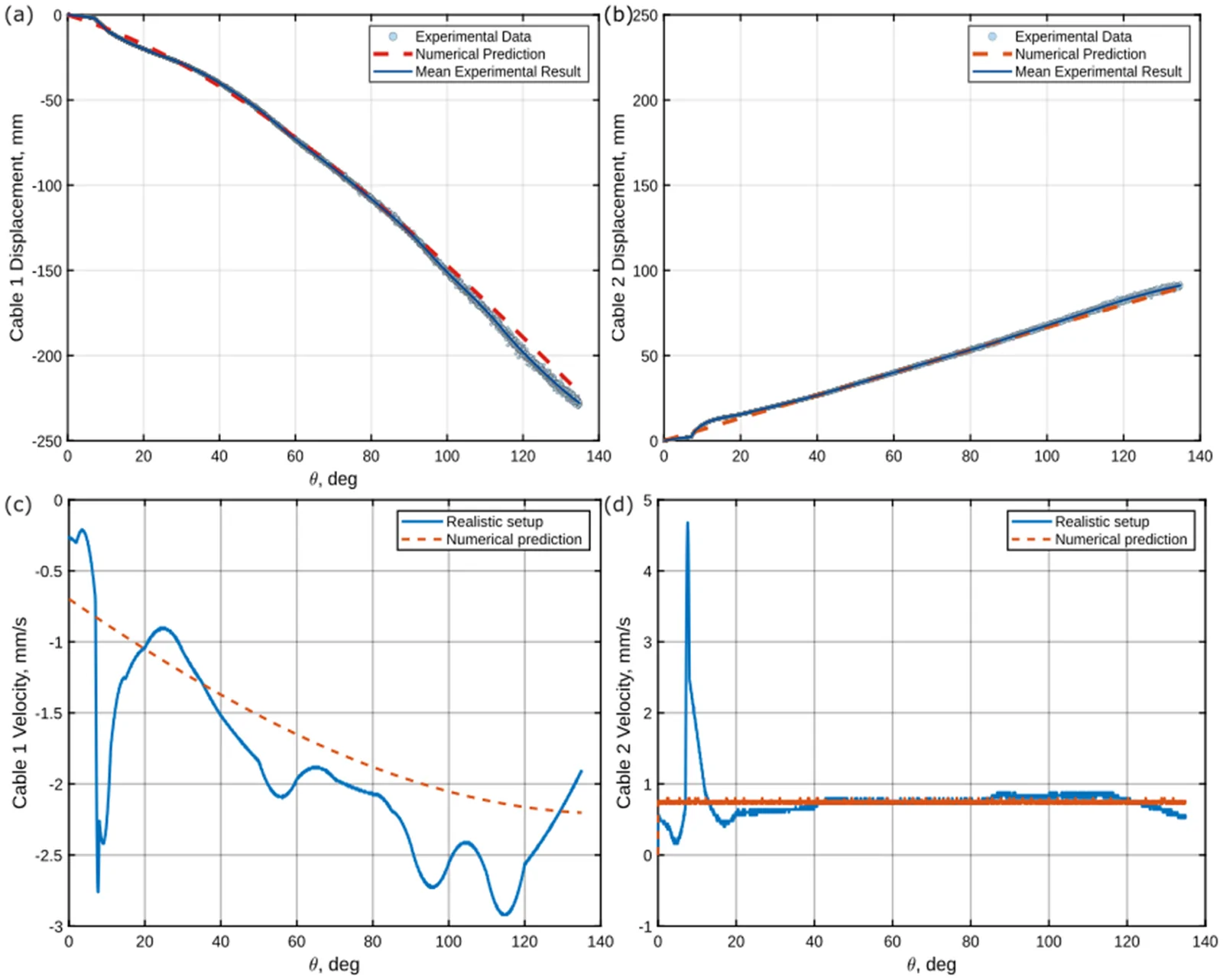
Kinematic model validation via cable displacement and velocity measurements. Comparative analysis of theoretical predictions against experimental data for (a) Cable 1 displacement; (b) Cable 2 displacement; (c) Cable 1 velocity, and (d) Cable 2 velocity across varying elbow joint angles (θ).

To quantitatively evaluate how closely the experimental cable displacement $\Delta l_{\text{exp},i}$ conforms to the analytical model prediction $\Delta l_{\text{num},i}$ across all sample points $N_{p}$, the Root Mean Square Error (RMSE) and Maximum Absolute Error ($\text{MA}E_{\text{max}}$), and Mean Error **(**$\mu_{e}$**)** are computed using Eq. (17):

(17) $$\text{RMSE}=\sqrt{\frac{1}{N_{p}}\sum_{i=1}^{N_{p}}{\left(\Delta l_{\text{exp},i}-\Delta l_{\text{num},i}\right)}^{2}},\;\text{MA}E_{\text{max}}=\max_{1\leq i\leq N_{p}}\left|\Delta l_{\text{exp},i}-\Delta l_{\text{num},i}\right|,\;\mu_{e}=\frac{1}{N_{p}}\sum_{i=1}^{N_{p}}\left(\Delta l_{\text{exp},i}-\Delta l_{\text{num},i}\right),\;i=1,2$$

For Cable 1 in Table 4, the mean experimental trajectory (average result of experimental cable displacement of all trials) follows the theoretical prediction, achieving an RMSE of 3.82 mm and a maximum absolute error of 8.92 mm near peak flexion. For Cable 2, the error remains minimal across the entire trajectory, resulting in an RMSE of 1.46 mm and a maximum absolute error of 3.56 mm. These results confirm that the inverse kinematic model reliably predicts actual cable travel under low-speed operation.

**Table 4:** Kinematic tracking statistical metrics ($0^{\circ }$ to $135^{\circ }$).

Cable	Metric	Trial 1	Trial 2	Trial 3	Trial 4	Trial 5	Mean of Trials	Mean Experimental Result
**Cable 1 (Flexion)**	Root Mean Square Error (RMSE)	4.09mm	4.17mm	4.2mm	4.1mm	4.13mm	4.13 mm ±0.04	3.82mm
	Maximum Absolute Error (Max|e|)	17.92mm	20.54mm	16.86mm	16.15mm	15.63mm	17.42 mm ±2.02	8.92mm
	Mean Error ($\mu_{e}$)	−1.92mm	−1.98mm	−1.99mm	−1.92mm	−1.98mm	(−1.96 ± 0.03)mm	−1.96mm
**Cable 2 (Extension)**	Root Mean Square Error (RMSE), mm	1.53mm	1.53mm	1.52mm	1.54mm	1.52mm	(1.53±0.01)mm	1.46mm
	Maximum Absolute Error (Max|e|)	4.23mm	5.03mm	4.71mm	4.28mm	4.99mm	(4.65±0.38)mm	3.56mm
	Mean Error ($\mu_{e}$)	0.89mm	0.87mm	0.87mm	0.89mm	0.87mm	(0.88 mm ±0.01)mm	0.88mm

To clarify the statistical metrics in Table 4, the Mean Experimental Result was obtained by first averaging the recorded cable displacement values from the 5 individual trials at each discrete time point to generate a single mean displacement profile, from which the overall RMSE, Max |e|, and $\mu_{e}$ were evaluated. In contrast, the column with Mean of Trials represents the simple arithmetic average of the calculated metric values across the 5 separate trials.

The mean error ($\mu_{e}$) was calculated in Eq. (17) by taking the arithmetic average of the discrete point-by-point tracking errors $e_{i}=\Delta l_{\text{exp},i}-\Delta l_{\text{num},i}$ across the entire dataset containing $N_{p}=2,401$ recorded data points per trial. The resulting mean error for Cable 1 displays a minor negative bias of $\mu_{e}=-1.96\;\text{mm}$. In contrast, Cable 2 exhibits a minimal mean error of $\mu_{e}=0.88\;\text{mm}$ due to its smaller total displacement compared to Cable 1.

### Static validation

To evaluate the structural behavior comprehensively, both the single-cable (Scenario 1) and dual-cable antagonistic (Scenario 2) configurations were tested separately. In the single-cable configuration, the second cable was fully released because gravity assists the platform toward the left cable, allowing Cable 1 to balance the gravitational load independently without requiring tension on Cable 2. Conversely, the dual-cable configuration incorporates Cable 2 as an opposing tendon to provide continuous pre-tension, eliminate slack, and keep the platform securely constrained throughout the entire range of motion.

#### Scenario 1: single-cable configuration

The experimental evaluation focused on mapping the force profiles under the two distinct loading and directional regimes defined in the theoretical framework, corresponding directly to the configurations illustrated in Fig. 9.

**Fig. 9:**
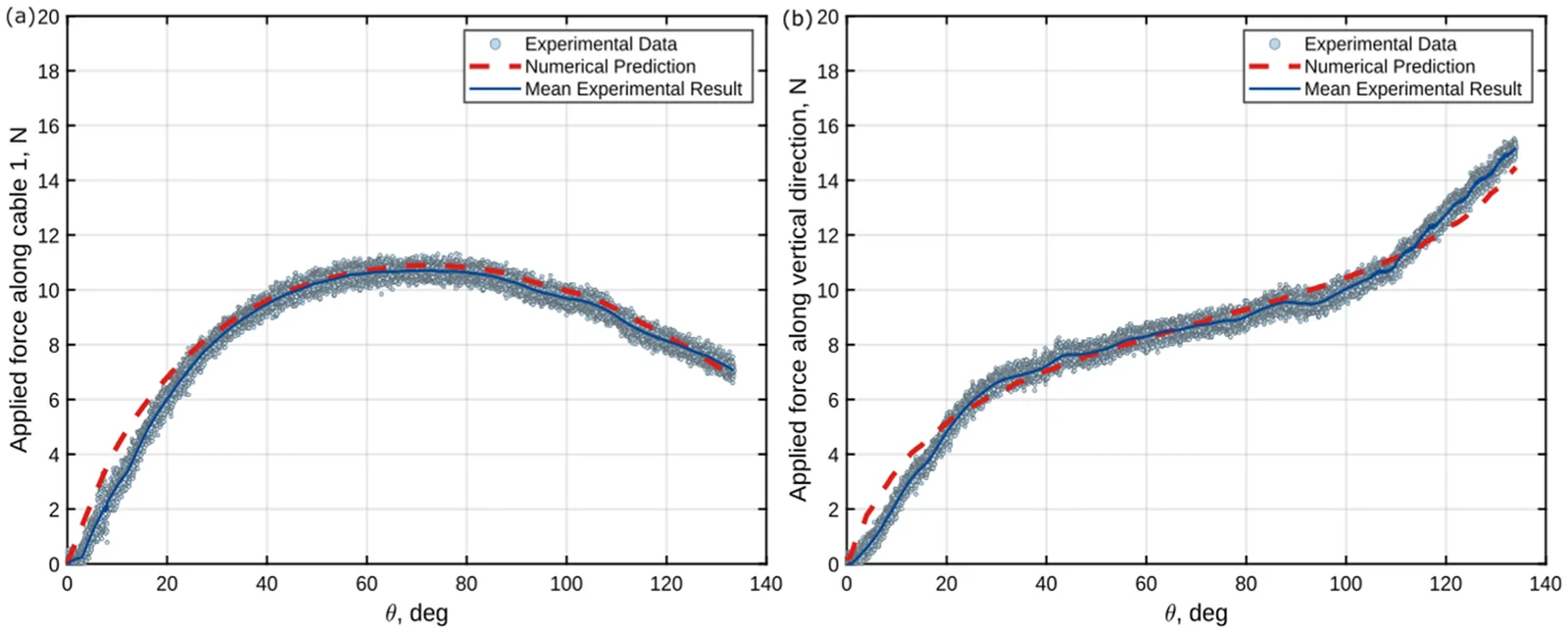
Comparison of theoretical and experimental static force profiles under geometric parameters h = 240 mm and D = 80 mm: (a) Pulling force measured along the Cable 1 line of action, and (b) force component tracked along the vertical direction of the moving platform relative to the cable attachment point.

Fig. 9a presents the applied pulling force tracking along Cable 1, while Fig. 9b depicts the force tracking component along the vertical direction of the moving platform. In both graphs, the experimental curves derived from the real prototype follow the theoretical predictions, confirming that the developed pseudo-static equilibrium and kinematic transformation models represent the load distribution and gravitational compensation of the system.

Regarding the deviation zones within the graphs, the larger discrepancies between the experimental prototype measurements and the theoretical predictions are concentrated primarily in the initial low-angle flexional zone (approximately $2^{\circ }$ to $17^{\circ }$ for Cable 1 and $3^{\circ }$ to $12^{\circ }$ for the vertical direction). Within this early transitional phase, the maximum absolute errors peak at 1.55N for Cable 1 and 1.34N for the vertical direction. For angles exceeding $20^{\circ }$, deviations drop significantly, reflecting stable and high-accuracy tracking across the higher operating range.

The accompanying summary table (Table 5) consolidates these findings across the five repeated trials, reporting a root mean square error (RMSE) of 0.51N for both Cable 1 and the vertical direction. The small signed mean errors ($\mu_{e}=-0.33\;N$ for Cable 1 and −0.08N for the vertical direction) indicate a negligible overall offset or systematic calibration bias between the theoretical model and the experimental measurements across the tested range of motion.

**Table 5:** Static force tracking error metrics ($0^{\circ }$ to $135^{\circ }$).

Cable	Metric	Trial 1	Trial 2	Trial 3	Trial 4	Trial 5	Mean of Trials	Mean Experimental Result
**Cable 1 (Flexion)**	Root Mean Square Error (RMSE)	0.37 N	0.78 N	0.52 N	0.51 N	0.52 N	(0.54±0.15)N	0.51N
	Maximum Absolute Error (Max|e|)	1.43 N	2.77 N	1.76 N	2.10 N	1.76 N	(1.96±0.51) N	1.55N
	Mean Error ($\mu_{e}$)	−0.02 N	−0.64 N	−0.33 N	−0.33 N	−0.33 N	(−0.33±0.22) N	−0.33N
**Cable 2 (Extension)**	Root Mean Square Error (RMSE)	0.51 N	0.52 N	0.63 N	0.55 N	0.52 N	(0.55±0.05)N	0.51N
	Maximum Absolute Error (Max|e|)	1.50 N	1.50 N	1.84 N	1.46 N	1.46 N	(1.55 ± 0.16) N	1.34N
	Mean Error ($\mu_{e}$)	−0.08 N	−0.08 N	−0.37 N	0.21 N	−0.08 N	(−0.08 ± 0.21) N	−0.08N

#### Scenario 2: dual-cable configuration

Fig. 10 presents the experimental static force profiles for the dual-cable antagonistic configuration (Scenario 2), where both cables are actively maintained under tension to keep the platform mechanically constrained throughout the motion. By following Algorithm 1, the tension force along each cable exceeds the minimum limit ($F_{\text{min}}=1\;N$) across the entire trajectory.

**Fig. 10:**
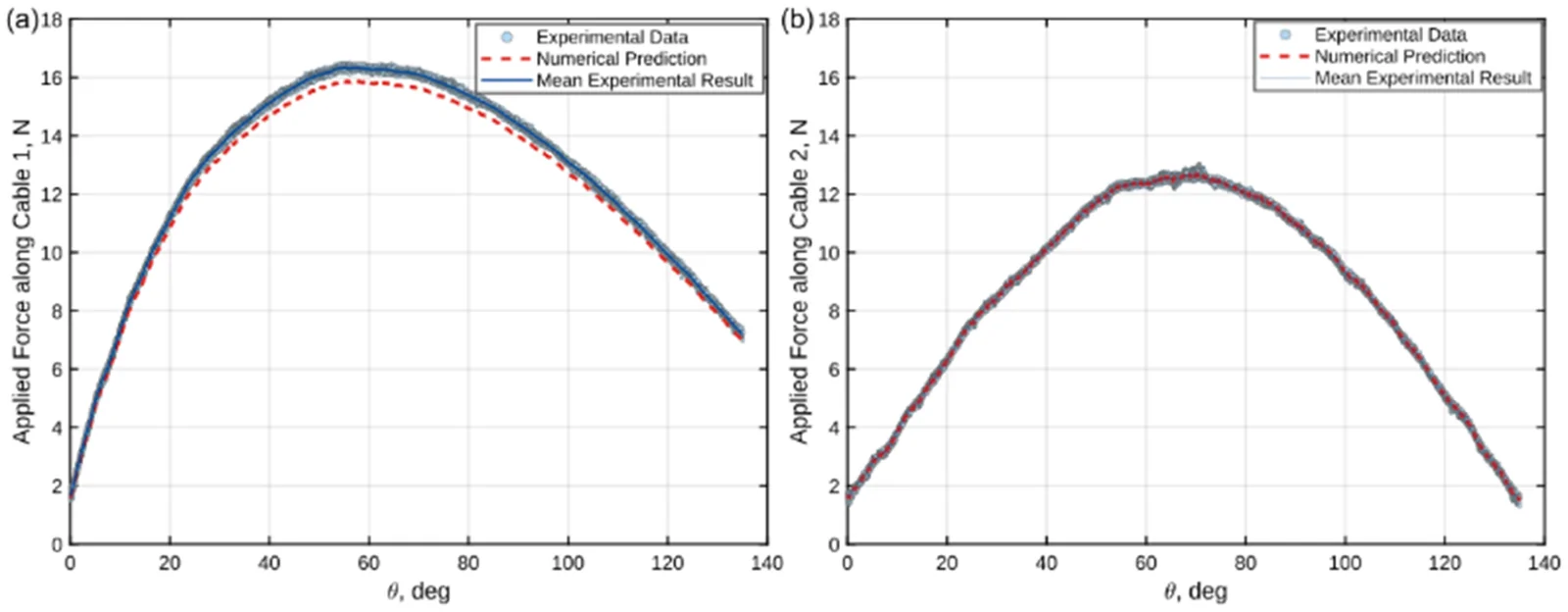
Experimental static force profiles for the dual-cable antagonistic configuration (Scenario 2) across the range of motion ($\theta =0^{\circ }$ to $135^{\circ }$): (a) Pulling force measured along Cable 1, and (b) concurrent tension force along Cable 2.

When comparing the pulling force along Cable 1 in dual-cable operation (Fig. 10a) to the single-cable setup (Fig. 9a), the overall behavioral trend is similar, with both curves following a smooth arch. However, a significant quantitative difference is present due to the second cable's opposed force. In Fig. 9a (Scenario 1, where the second cable was fully released), Cable 1 reaches a peak force of approximately 11N around $\theta =70^{\circ }$. In contrast, in Fig. 10a (Scenario 2, where both cables were tensioned), the tension along Cable 1 is substantially higher, exhibiting the behavioral profile predicted by the single-cable configuration—following the expected curved trajectory to a peak of approximately 16N near $\theta =70^{\circ }$ before dropping to roughly 7.5N at the maximum flexion angle of $\theta =135^{\circ }$.

As illustrated in Fig. 10b, the second cable is tensioned concurrently throughout the motion because the first cable acts as a counterbalance force. The measured $F_{2}\left(\theta \right)$ dataset follows the dual-cable static model predictions, mirroring a similar curved profile that starts from a controlled pre-load baseline of roughly 2N at $0^{\circ }$, peaks at approximately 13N around $\theta =70^{\circ }$, and decreases back to about 1.5N near $\theta =135^{\circ }$. This quantitative distribution confirms that Cable 2 successfully provides continuous antagonistic constraint and gravity-assisted balancing throughout the entire operational workspace without encountering slack.

The mean experimental force profile of Cable 2 was obtained by averaging the measured tension across the five trials and was used as an experimental input to Eq. (13). Then Eq. (13) was evaluated to predict the corresponding Cable 1 tension, which was independently compared with the measured Cable 1 force profiles from each of the five trials, yielding trial-specific metrics including Root Mean Square Error (RMSE), Maximum Absolute Error (Max|e|), and Mean Error ($\mu_{e}$). Therefore, the quantitative model-validation metrics reported for Scenario 2 primarily evaluate the correspondence between the predicted and experimentally measured Cable 1 forces. Cable 2 measurements represent the experimentally measured antagonistic input to the two-cable equilibrium formulation rather than an independently predicted output.

As detailed in Table 6, averaging these individual trial evaluations produced the Mean of Trials metrics for Cable 1, resulting in an RMSE of 0.39 N, a Maximum Absolute Error of 0.67 N, and a Mean Error of 0.37 N. Furthermore, comparing the overall point-by-point mean experimental Cable 1 force profile against the numerical prediction yielded an RMSE of 0.38 N, a Maximum Absolute Error of 0.48 N, and a Mean Error of 0.36 N. For Cable 2, the metrics reported in Table 6 characterize the repeatability and deviation of the individual experimental trials relative to the mean experimental input used in Eq. (13). The close alignment between the numerical prediction and measured experimental trajectories for Cable 1 confirms the validity and reliability of the dual-cable static model.

**Table 6:** Peak cable forces, mean values, and absolute force tracking errors across experimental trials.

Cable	Metric	Trial 1	Trial 2	Trial 3	Trial 4	Trial 5	Mean of Trials	Mean Experimental Result
**Cable 1 (Flexion)**	Root Mean Square Error (RMSE)	0.39 N	0.42 N	0.37 N	0.37 N	0.38 N	(0.39 ± 0.02) N	0.38N
	Maximum Absolute Error (Max|e|)	0.73 N	0.67 N	0.68 N	0.64 N	0.64 N	(0.67 ± 0.04) N	0.48N
	Mean Error ($\mu_{e}$)	0.37 N	0.4 N	0.35 N	0.35 N	0.36 N	(0.37 ± 0.02) N	0.36N
**Cable 2 (Extension)**	Root Mean Square Error (RMSE)	0.09 N	0.068N	0.061 N	0.058N	0.058 N	(0.067 ± 0.013) N	N/A (Baseline Input)
	Maximum Absolute Error (Max|e|)	0.36 N	0.22N	0.2 N	0.22N	0.21 N	(0.242 ± 0.07)N	N/A (Baseline Input)
	Mean Error ($\mu_{e}$)	−0.008 N	0.034N	−0.016N	−0.006N	−0.005N	0 ± 0.020	N/A (Baseline Input)

### Quantitative performance benchmarks

Quantitative performance assessment. Prototype fidelity was assessed quantitatively using the root mean square error (RMSE), maximum absolute error, and mean error between the analytical predictions and experimental measurements. These metrics were evaluated separately for cable displacement and static force tracking across five repeated trials. Trial-specific results together with the mean ± standard deviation are reported to characterize both model–experiment relationship and experimental repeatability.

### Limitations

The differences seen between the theoretical model and the physical prototype primarily stem from inherent mechanical imperfections within the assembly. Primarily, localized mechanical friction generated as the cables rub against the 3D-printed structural components and pass through intermediate routing channels causes a slight upward shift in the measured force during active pulling phases. This effect is compounded by mechanical backlash between the shaft and gear of the central elbow joint, as well as within the motor transmission gears, which introduces micro-displacements before the physical load registers on the sensors.

Additionally, geometric irregularities in the cable winding shafts introduce low-amplitude fluctuations; because the 2.5-mm steel cable does not wrap in a perfectly uniform plane, small variations occur in the effective spool radius and subsequent mechanical advantage. Minor deviations also arise from manufacturing tolerances of the 3D-printed PLA-based components. A slight force sensor bias, combined with the smoothing action of the MATLAB/Simulink median filter used to suppress high-frequency noise, accounts for the dampened nature of the experimental curve relative to the sharper analytical line.

Additionally, the Bowden cable routing mechanism for the opposing cable is treated analytically as a nominal, length-invariant guide that transmits displacement. In physical implementations, curvature-dependent friction, internal clearance, backlash, and sheath compliance affect displacement and tension transmission. Crucially, because cable displacement in this study was inferred directly from the motor encoder and spool geometry, the current validation measures motor-side cable travel rather than direct distal displacement at the joint. Although these distributed effects were partially mitigated by inline force feedback filtering, unmodeled structural and Bowden friction contribute to the minor tracking discrepancies observed between theoretical predictions and experimental results. Because independent distal position sensing (optical motion tracking or external joint encoders) was not implemented in this prototype, these results should be interpreted as motor-side cable-travel validation. Future iterations of this work will incorporate independent distal tracking sensors as well as rigorous input-output displacement and tension calibration protocols to explicitly quantify and compensate for these transmission losses.

While the platform diameter is kept constant at D=80 mm, future work will evaluate the system across varying platform diameters. A limitation of the current fixed-scale prototype (D=80mm) is that choosing a cable holder radius smaller than half the platform diameter (r<D/2) introduces physical wall and structural link collisions. While smaller values of r are mathematically viable for cable stroke minimization, they require a fully variable multi-parameter co-design (scaling the platform diameter and link geometry concurrently) rather than independent radius selection. Consequently, r=40mm was established as the practical lower boundary for the current physical architecture.

In the current implementation, antagonistic co-contraction is indirectly bounded by the hysteresis release threshold ($F_{\text{upper}}=1.2\;N$) and enforced by hardware-level motor current limits and over-tension safety cutoffs ($F_{\text{max}}$). While the master–slave coordination rule prevents actuator fighting during simultaneous low-tension events, active real-time adjustment of internal co-contraction forces was not explicitly incorporated into the high-level trajectory controller. Future work will focus on integrating continuous force-balancing feedback loops to actively adjust antagonistic tension bias without relying on discrete length corrections.

Finally, the present force model represents the robotic mechanism only; human forearm/hand mass, passive joint impedance, and active muscular torques are not included and would need to be incorporated before extending the model to human-assistance conditions.

## Ethics statements

The experimental validation reported in this article was performed on the mechanical prototype itself and did not involve human participants, human tissue or samples, animal experiments, or data collected from social media platforms.

## CRediT author statement

**Adilet Toleshev:** Writing – original draft, Investigation, Visualization. **Sharafatdin Yessirkepov:** Formal analysis, Conceptualization. **Aibek Niyetkaliyev:** Methodology, Supervision, Writing – review & editing. **Ali Kaiyrdykozha:** Software, Hardware. **Aiman Ozhikenova:** Validation, Writing – review & editing. **Lashin Bazarbay:** Methodology, Validation. **Ozhiken Assylbek:** Project administration, Investigation, Supervision. **Igembay Yerbolat:** Visualization, Software.

## Related research article

None.

## For a published article

None.

Supplementary material *and/or* additional information [Optional]

CAD files, MATLAB/Simulink models, and raw experimental data associated with this study are available at https://doi.org/10.5281/zenodo.23169245

## Declaration of competing interest

The authors declare that they have no known competing financial interests or personal relationships that could have appeared to influence the work reported in this paper.

## Data Availability

Data will be made available on request.
